# Percentile Curves for Multiple Physical Fitness Components Among Chinese Han Children and Adolescents Aged 7–18 Years From a National Survey Based on the Total and the Normal Weight Population

**DOI:** 10.3389/fnut.2021.770349

**Published:** 2022-01-03

**Authors:** Ning Ma, Jiajia Dang, Yunfei Liu, Panliang Zhong, Xiaojin Yan, Jingshu Zhang, Yanhui Dong, Yi Song, Jun Ma, Patrick W. C. Lau

**Affiliations:** ^1^Institute of Child and Adolescent Health, School of Public Health, Peking University, Beijing, China; ^2^National Health Commission Key Laboratory of Reproductive Health, Beijing, China; ^3^Department of Sport, Physical Education & Health, Hong Kong Baptist University, Kowloon Tong, Hong Kong SAR, China; ^4^Laboratory of Exercise Science and Health, BNU-HKBU United International College, Zhuhai, China

**Keywords:** physical fitness, percentiles, children and adolescents, normal weight population, China, sex difference

## Abstract

**Introduction:** To develop sex- and age-specific percentile curves for seven physical fitness components for Chinese Han children and adolescents aged 7–18 years based on the total and the normal weight population using a nationally representative sample.

**Methods:** A total of 214,228 Chinese Han children and adolescents aged 7–18 years old with all nutritional status and 161,999 with normal weight were examined. Seven physical fitness components [forced vital capacity (FVC), standing long jump (SLJ), 50-m dash, sit-and-reach (SR), grip strength (GS), body muscle strength (BMS), and endurance running (ER)] were measured, and percentile curves for each physical fitness component at the 20th, 40th, 60th, and 80th percentiles were calculated using the general additive model for location, scale, and shape (GAMLSS).

**Results:** Physical fitness presents different characteristics in each subgroup of sex, age, and nutritional status among children and adolescents. Sex- and age-specific percentiles for the seven physical fitness components among the Chinese Han children and adolescents aged 7–18 years based on the total and the normal weight population were provided as curves. Boys performed better than girls in FVC, SLJ, 50-m dash, GS, and ER but worse in SR. The performances of FVC, SLJ, 50-m dash, GS, BMS, and ER increased with age, but the estimates of SR were at the bottom among boys aged 12 years and girls aged 11 years. The annual increments of all components were larger in boys than girls at the peak time, which was earlier in girls than boys. The gap of physical fitness components between sexes increased with age, especially during puberty (since after 11 years old).

**Conclusion:** The present study described the percentile curves of seven physical fitness components among the Chinese Han children and adolescents based on the total and the normal weight population at the national level, which could help to chart the level of physical fitness across age span and identify the extreme populations with either health concerns or potential talents.

## Introduction

Physical fitness is the ability of an individual to accomplish daily tasks with vigor and alertness, without undue fatigue and with ample energy to enjoy leisure-time pursuits, and to meet unforeseen emergencies (1) and is usually considered an integrated measure of most body functions in the performance of daily physical activity (2). Meanwhile, many studies have discovered meaningful associations between physical fitness and health in both childhood and adulthood (3), i.e., higher physical fitness level is associated with more favorable health outcomes, including reducing the risk of obesity (4, 5) and cardiovascular diseases (6, 7), as well as promoting skeletal health (8) and mental health (9, 10).

Normative-referenced percentile evaluation is one of the most common methods to assess physical fitness, providing the relative position of an individual's physical fitness level within a group (12). It can be used to identify individuals with low performance in need of primary prevention, as well as helping in health promotion policies, and to identify high-performing individuals as part of a sports talent identification program (2, 13, 14). Consequently, many countries have developed normative-referenced percentile values for children and adolescents with different age ranges in one or more physical fitness components (12, 15–17). In China, national standards for assessing the physical fitness of children and adolescents have been established (11). However, these standards provide scores only for each physical fitness component, which might cause children and adolescents, especially those with lower levels, to overestimate their physical fitness; for example, the score of 60 usually corresponds to P_10_ of the reference population for each physical fitness component. Moreover, few recent studies have produced normative-referenced percentile values for a comprehensive set of physical fitness tests in China (18).

Moreover, many studies have confirmed the relationship between nutritional status and physical fitness, in which children and adolescents with normal weight have a higher level of physical fitness than those thin, overweight, or obese (19, 20), and China is in a phase of nutritional and lifestyle transition with looming burden of overweight and obesity, the percentiles of physical fitness among Chinese children and adolescents during the past two decades are likely to shift downward, and this trend might continue (19). In previous studies, the percentile values have always been established based on the total sample, however, it might be unduly influenced by the proportion of the overweight and obese population. Therefore, the percentile values based on the normal weight population might provide different cutoffs, which might help identify more children and adolescents with poor physical fitness.

We hypothesize that physical fitness demonstrates different characteristics in each subgroup by nutritional status among children and adolescents. In this study, we aimed to establish the age- and sex-specific percentile curves of the seven physical fitness components among the total and the normal weight population of Chinese children and adolescents aged 7–18 years old by using the national representative data. Considering the differences in physical fitness performance from the different ethnic groups (21, 22), only children and adolescents from Han, the dominant ethnic group in China, were selected in this study.

## Methods

### Study Design and Participants

**Figure 1:**
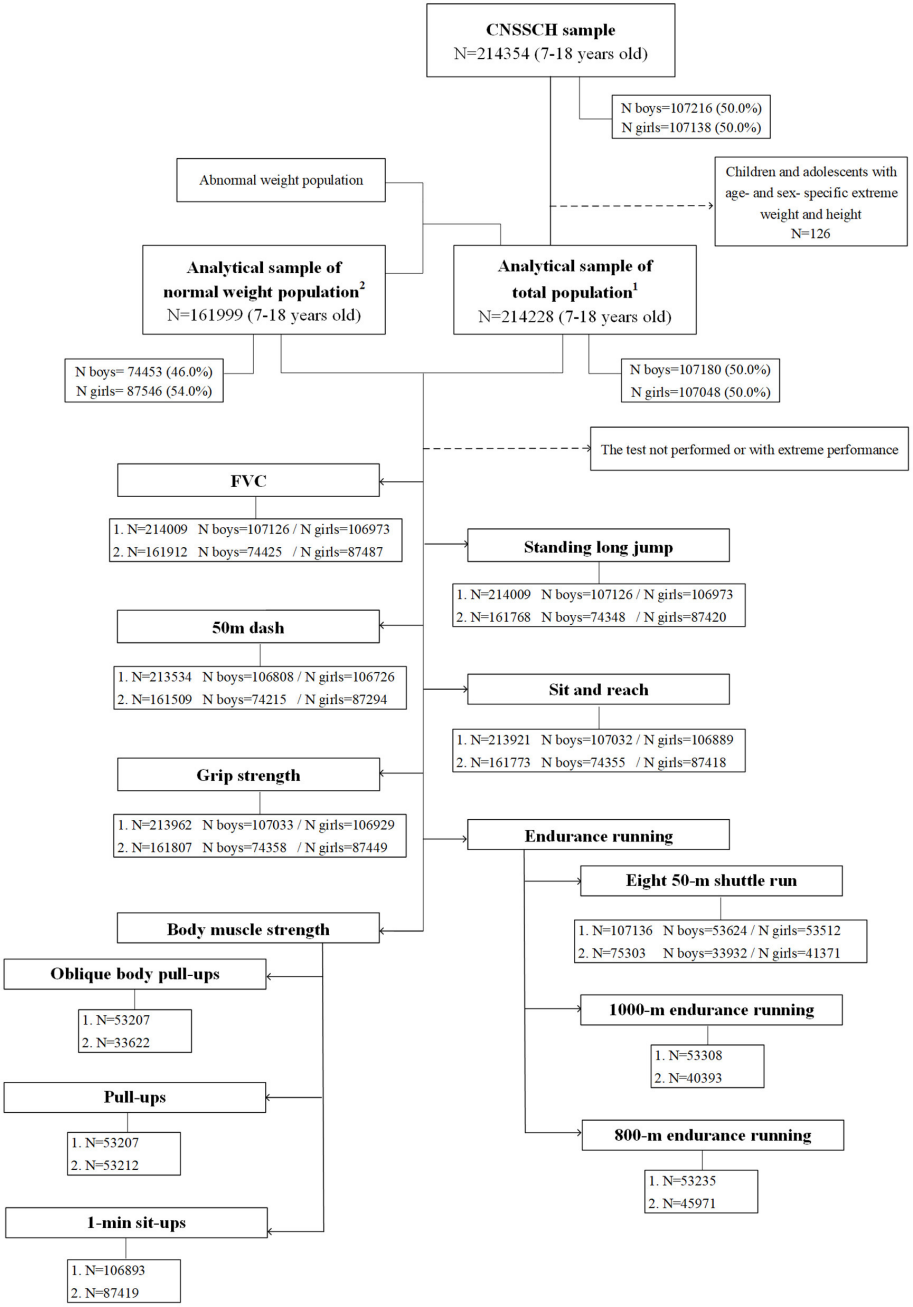
Flowchart of the population involved in this study from the 2014 Chinese National Survey on Students' Constitution and Health (CNSSCH) study.

Data were obtained from the 2014 Chinese National Survey on Students' Constitution and Health (CNSSCH). The CNSSCH was the largest nationally representative survey of the physical fitness of Chinese children and adolescents aged 7–18 years with a nature of cross-sectional study design, which has been conducted on a regular basis once every 5 years since 1985. It used a multistage stratified cluster sampling design as described in the previous studies (23). That is, three prefecture-level cities were first selected according to their socioeconomic status in each province. Then, the children and adolescents from these three cities were stratified by urban and rural areas based on their residence location, within each stratified area, schools covering students aged 7–18 years were randomly selected in 1985 and the framework of selected schools kept uniform subsequently. At last, from these schools, all students from the randomly selected classes by grade were included in the 2014 CNSSCH. Briefly, it covered 31 provinces except Hong Kong, Macau, and Taiwan. This study included only participants of Han ethnicity, who account for 92% of the total Chinese population from 26 provinces and four municipalities of mainland China, excluding Tibet (where the Han is a minority). About 126 (0.06%) participants with missing or biologically implausible data on anthropometric measurements were excluded, and a total of 214,228 participants (107,180 boys and 107,048 girls) were included in the analysis based on the total population. The nutritional status of children and adolescents is shown in Supplementary Table 1, after excluding 24.4% of participants with abnormal weight (such as, thinness, overweight, and obesity), 161,999 participants (74,453 boys and 87,546 girls) with normal weight were further analyzed. Since not all children and adolescents participated in all the tests, sample sizes of subgroups vary for the different physical fitness components (Figure 1). This study was approved by the Medical Research Ethics Committee of the Peking University Health Science Center (IRB00001052-18002), Beijing, China.

### Anthropometric Measurements

Height (centimeters) and weight (kilogram) were measured by trained staff following a standardized CNSSCH procedure. While measuring, the participants were stood erect, barefoot, and at ease with only light clothing. Height was measured with a portable stadiometer to the nearest 0.1 cm, and weight was recorded in units of 0.1 kg with a standardized scale using the mean of three measurements. Both stadiometers and scales were calibrated before use and similar instruments were used at each survey site according to the requirement of the 2014 CNSSCH survey protocol. BMI was calculated as body weight (kilogram) divided by height (meters) squared (kilogram per square meter). BMI-for-age Z-score (BAZ) was calculated with the WHO 2007 references, and the reference population used here was the fixed population recommended by the WHO (24). Thinness and overweight (i.e., obesity) were defined by using the growth references of BAZ: thinness: < −2 SD, overweight: > + 1 SD (25).

### Physical Fitness Tests

Seven physical fitness components [forced vital capacity (FVC), standing long jump (SLJ), 50-m dash, sit-and-reach (SR), grip strength (GS), body muscle strength (BMS), and endurance running (ER)] were measured by a team of trained field investigators following the standardized procedures. FVC, SLJ, 50-m dash, SR, and GS were measured across age of 7–18 years. BMS was assessed by oblique body pull-ups (boys aged 7–12 years), pull-ups (boys aged 13–18 years), and 1-min sit-ups (girls aged 7–18 years). ER was assessed by eight 50-m shuttle runs (both boys and girls aged 7–12 years), 1,000-m ER (boys aged 13–18 years) and 800-m ER (girls aged 13–18 years).

The protocols used for physical fitness testing are described in detail below (some details for SR, oblique body pull-ups, and pull-ups are shown in Supplementary Figures 1–3).

The FVC test was measured to assess cardiorespiratory fitness of children and adolescents (26). Participants were commanded to try their best to inhale deeply to their limits and then exhale slowly until they could no longer breathe out. Its performance was recorded to the nearest 1 ml with a spirometer using the maximum of two measurements.

The SLJ test was used to evaluate lower body explosive power (27). Participants were required to stand behind the take-off line with feet placed parallel at shoulder width. Arm swinging before the jump was allowed. The distance was measured from the take-off line to the nearest contact with the floor landing with both feet together. The maximum value of three measurements was recorded to the nearest 1 cm.

The 50-m dash test was used to assess the speed performance of children and adolescents (17, 28). Standing in the frontal erect posture with their feet behind the starting line, participants ran on the whistle and accelerated with maximum effort. The time from the starting line to the finish line was recorded to the nearest 0.1 s.

The SR test assessed the flexibility of children and adolescents. Participants sat on the floor with their legs fully extended in front and feet without shoes placed on the baffle of the testing instruments. Bending their upper body and with the palms facing down and hands by the side, they were asked to extend forward as far as possible. Participants would get a score of 0 cm when their fingertips were aligned with the toe. The maximum value of the two measurements was recorded to the nearest 0.1 cm.

The GS test was used to assess the maximal isometric upper body muscular strength. Participants were required to stand upright with feet shoulder-width apart and arms in complete extension holding the dynamometer, and then, to squeeze the dynamometer as much as possible. The maximum value of the two measurements was recorded to the nearest 0.1 kg.

The oblique body pull-ups test reflected the strength and endurance of upper limb muscles. Holding the bar and stretching their legs forward, participants were required to maintain their arms at right angles to the torso and hang their body obliquely and then, do a curved arm pull-up. When the lower jaw could touch or exceed the bar, the arm was extended.

The pull-ups test was used to assess the strength and endurance of upper limb muscles. The participants faced the horizontal bar, stood naturally, and then, leaped up to hold the bar with the body in a straight arm hanging posture. After the body stopped swinging, it was pulled up with both arms at the same time. When the lower jaw exceeded the upper edge of the bar, the body was lowered to a straight arm hanging posture.

The 1-min sit-ups test was used to assess abdominal muscle strength and endurance (27, 29). Lying on a mat and bending knees at right angles with feet flat on the floor and held down by a partner, participants were required to sit up with both elbows touching or exceeding both knees to complete a test. The number of completions in 1 min was recorded.

The eight 50-m shuttle runs assess the cardiorespiratory endurance. With a standing start, after hearing the start signal, participants were required to run to the finish line, go around the benchmark in a counterclockwise direction, and then, run to the return line. The time for completing the above steps four times was recorded to the nearest 0.1 s.

The 1,000- and 800-m ER tests were used to evaluate the cardiorespiratory endurance. Participants were required to run to the finish line immediately with a standing start after hearing the start signal. The time from the starting line to the finish line was recorded to the nearest 0.1 s.

### Statistical Analysis

Descriptive statistics [median and inter-quartile range (IQR)] for boys and girls among the total population and normal weight population were all calculated. Comparisons between sexes among the two studies population were performed using the Mann–Whitney *U*-test. Percentile values for each physical fitness component among the total and the normal weight population were calculated using the general additive model for location, scale, and shape (GAMLSS) package (version 5.2-0) with age as a covariate stratified by sex. GAMLSS is an extension of the Lambda-Mu-Sigma (LMS) method and it establishes models through four parameters of distributions: median, coefficient of variation, skewness, and kurtosis. Box-Cox power exponential (BCPE), Box-Cox power exponential orig. (BCPEo), Box-Cox t (BCT), and Box-Cox t orig. (BCTo) distributions were fitted to each fitness outcome variable stratified by sex. P-splines were used to smooth the age trend for each fitness outcome using the generalized Akaike information criterion (GAIC). The Bayesian information criterion (BIC) was used to assess the goodness of fit to compare models. Worm plots and Q-Q plots were used for visual inspection (Supplementary Figures 4–12). The models providing the best balance between goodness of fit and smoothness were selected. For the selected models, the 5th, 10th, 20th, 30th, 40th, 50th, 60th, 70th, 80th, 90th, and 95th percentile were calculated for each physical fitness component. Given that GAMLSS does not work with zero or two values, we added a constant value (1 and 30 cm, respectively), in the components of 1-min sit-ups and SR (those three indicators with 0 score or negative scores) before model establishment and then reversed back by subtracting the same constant from the percentile results after the estimation of percentile values. Furthermore, we estimated percentile values for pull-ups using non-parametric estimates (i.e., the percentiles of observed distribution) considering the distribution of data. All statistical analyses were conducted in SPSS 26.0 (SPSS Inc, Chicago, IL, USA) and R (version 4.0.2).

## Results

Anthropometric characteristics and the physical fitness performance of Chinese Han children and adolescents based on the total and the normal weight population have been shown in Table 1. Overall, there was no age difference between sexes of the total population. Either among the total or normal weight population, the height and weight were significantly higher among boys than girls (*P* < 0.001), and physical fitness performances were better among boys in FVC, SLJ, 50-m dash, GS, and eight 50-m shuttle runs, while better among girls in SR across all ages (*P* < 0.001). When compared with the total sample, the median age of the normal weight population was older (*P* < 0.001) as abnormal weight was distributed unevenly across age groups (Supplementary Table 1). Both above indicated the need for sex- and age-specific analysis.

**Table 1:** Anthropometric characteristics and physical fitness performance of total and normal weight Chinese Han children and adolescents aged 7–18 years [median (interquartile range, IQR)].

	**Total population**			**Normal weight population**		
	**Boys**	**Girls**	***P*-Values**	**Boys**	**Girls**	***P*-Values**
Age (years)	12 (9, 15)	12 (9, 15)	0.962	13 (10, 16)	13 (10, 16)	<0.001
Height (cm)	157.5 (139.1, 169.5)	153.1 (138.9, 159.7)	<0.001	159.5 (138.7, 170.0)	153.5 (139.0, 160.0)	<0.001
Weight (kg)	47.5 (33.3, 58.8)	43.8 (32.0, 51.5)	<0.001	46.0 (31.1, 56.7)	43.3 (31.0, 50.3)	<0.001
BMI (kg/m^2^)	18.7 (16.6, 21.4)	18.4 (16.2, 20.7)	<0.001	17.8 (16.1, 19.7)	18.0 (16.0, 19.9)	0.026
Forced vital capacity (ml)	2,355 (1,557, 3,361)	1,880 (1,349, 2,412)	<0.001	2,428 (1,558, 3,408)	1,892 (1,353, 2,415)	<0.001
Standing long jump (cm)	175 (143, 213)	150 (131, 168)	<0.001	185 (150, 220)	152 (134, 170)	<0.001
50-m dash (s)	8.8 (7.7, 9.9)	9.9 (9.2, 10.7)	<0.001	8.5 (7.6, 9.7)	9.8 (9.2, 10.6)	<0.001
Sit-and-reach (cm)	7.0 (2.5, 11.7)	11.2 (6.8, 15.7)	<0.001	7.4 (3.0, 12.1)	11.4 (6.9, 16.0)	<0.001
Grip strength (kg)	24.4 (14.9, 37.4)	19.6 (13.2, 24.7)	<0.001	26.4 (14.9, 38.3)	19.8 (13.3, 24.7)	<0.001
Oblique body pull-ups	20 (12, 31)	–	–	22 (14, 33)	–	–
Pull-ups	2 (1, 5)	–	–	3 (1, 6)	–	–
1-min sit-ups	–	28 (21, 35)	–	–	28 (21, 35)	–
Eight 50-m shuttle runs (s)	122.8 (112.2, 135.0)	127.2 (117.6, 138.2)	<0.001	119.8 (110.0, 130.8)	125.8 (116.6, 136.6)	<0.001
1,000-m endurance running (s)	267.7 (245.1, 297.0)	–	–	262.3 (241.6, 289.2)	–	–
800-m endurance running (s)	–	257.0 (237.8, 280.7)	–	–	255.0 (236.3, 278.0)	–

**Figure 2:**
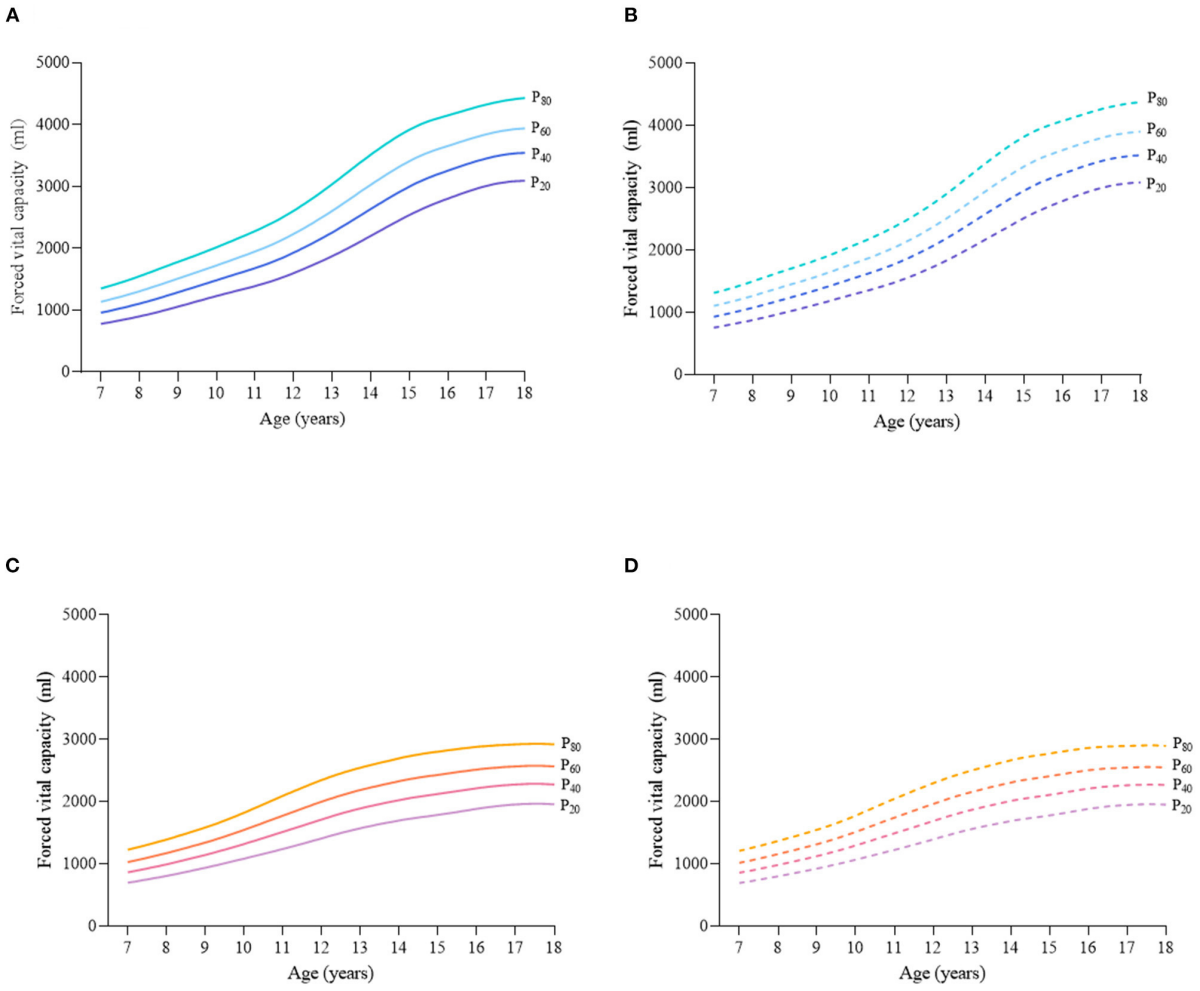
Sex- and age-specific percentile curves (P_20_, P_40_, P_60_, and P_80_) for forced vital capacity (FCV) (ml) for Chinese children and adolescents aged 7–18 years based on total population and normal weight population. **(A)** Boys-Total population, **(B)** Boys-Normal weight population, **(C)** Girls-Total population, and **(D)** Girls-Normal weight population.

**Figure 3:**
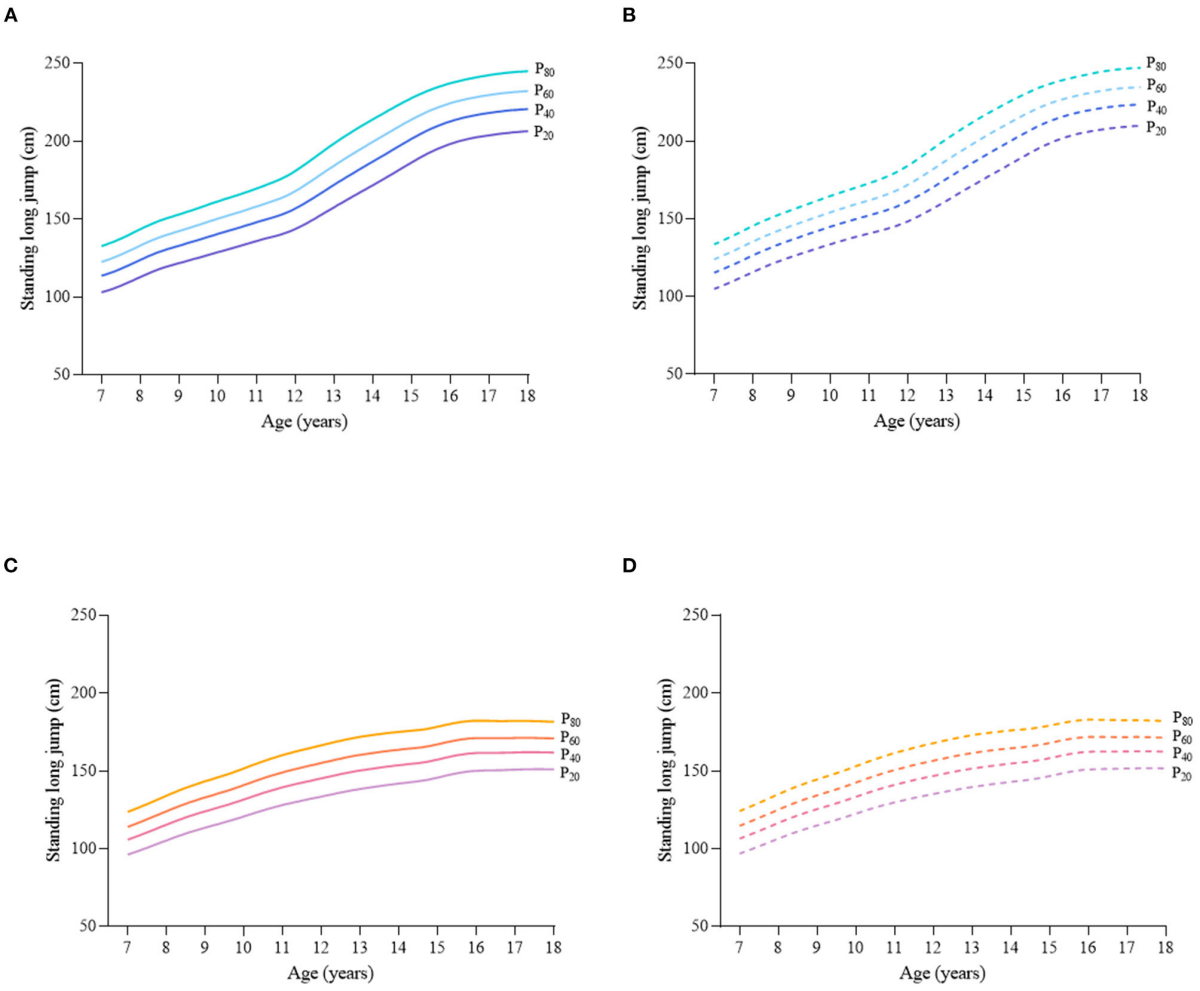
Sex- and age-specific percentile curves (P_20_, P_40_, P_60_, and P_80_) for standing long jump (SLJ) (cm) for the Chinese children and adolescents aged 7–18 years based on total population and normal weight population. **(A)** Boys-Total population, **(B)** Boys-Normal weight population, **(C)** Girls-Total population, and **(D)** Girls-Normal weight population.

**Figure 4:**
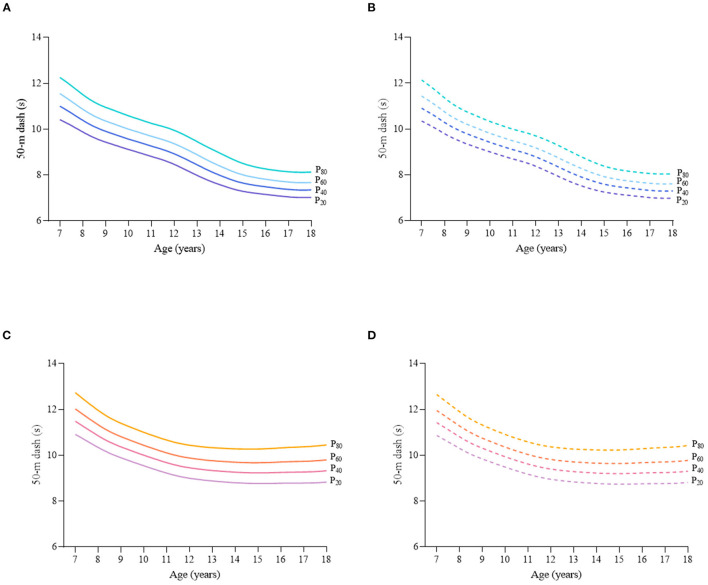
Sex- and age-specific percentile curves (P_20_, P_40_, P_60_, and P_80_) for 50-m dash (s) for the Chinese children and adolescents aged 7–18 years based on total population and normal weight population. **(A)** Boys-Total population, **(B)** Boys-Normal weight population, **(C)** Girls-Total population, and **(D)** Girls-Normal weight population.

**Figure 5:**
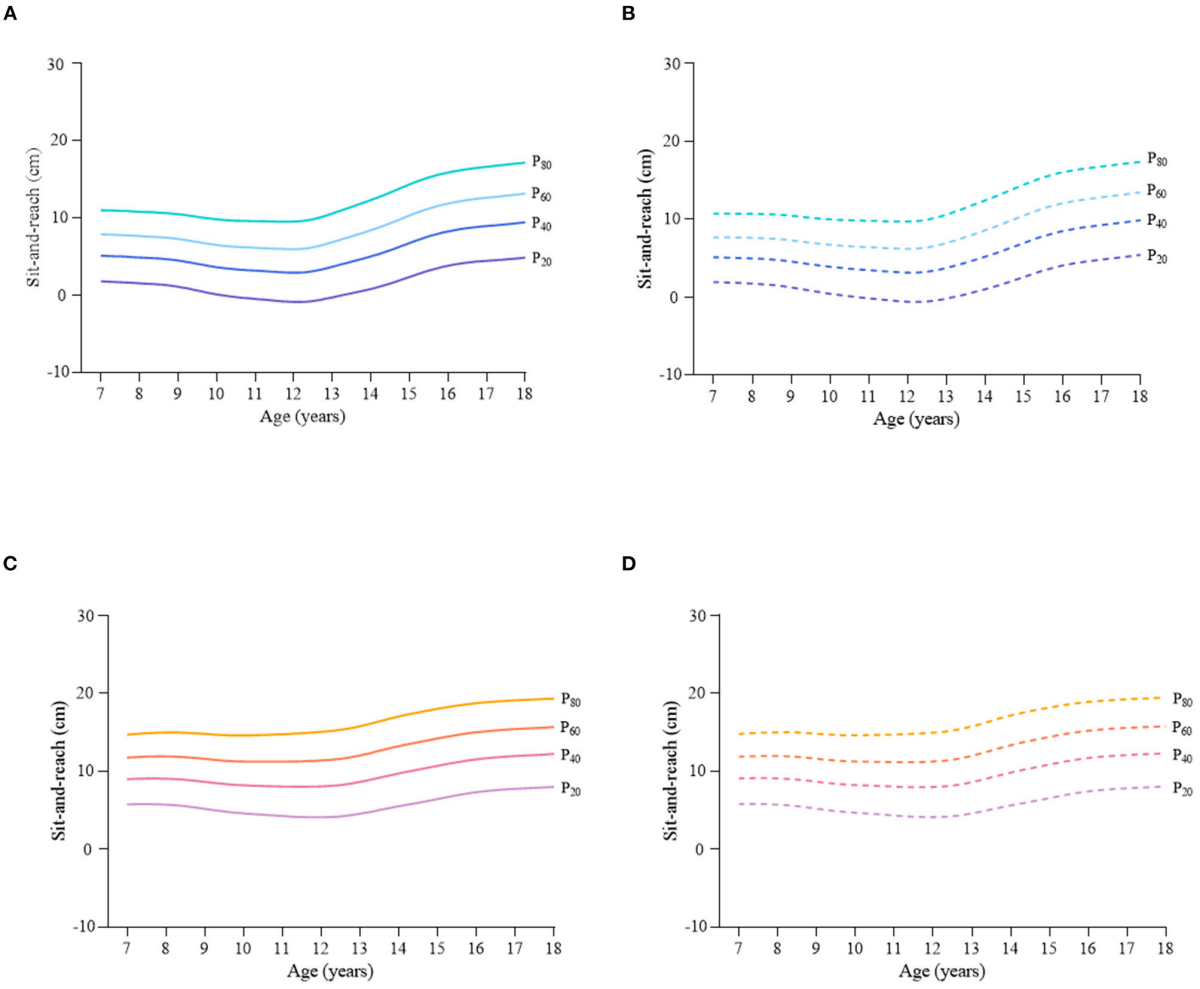
Sex- and age-specific percentile curves (P_20_, P_40_, P_60_, and P_80_) for sit-and-reach (SR) (cm) for the Chinese children and adolescents aged 7–18 years based on total population and normal weight population. **(A)** Boys-Total population, **(B)** Boys-Normal weight population, **(C)** Girls-Total population, and **(D)** Girls-Normal weight population.

**Figure 6:**
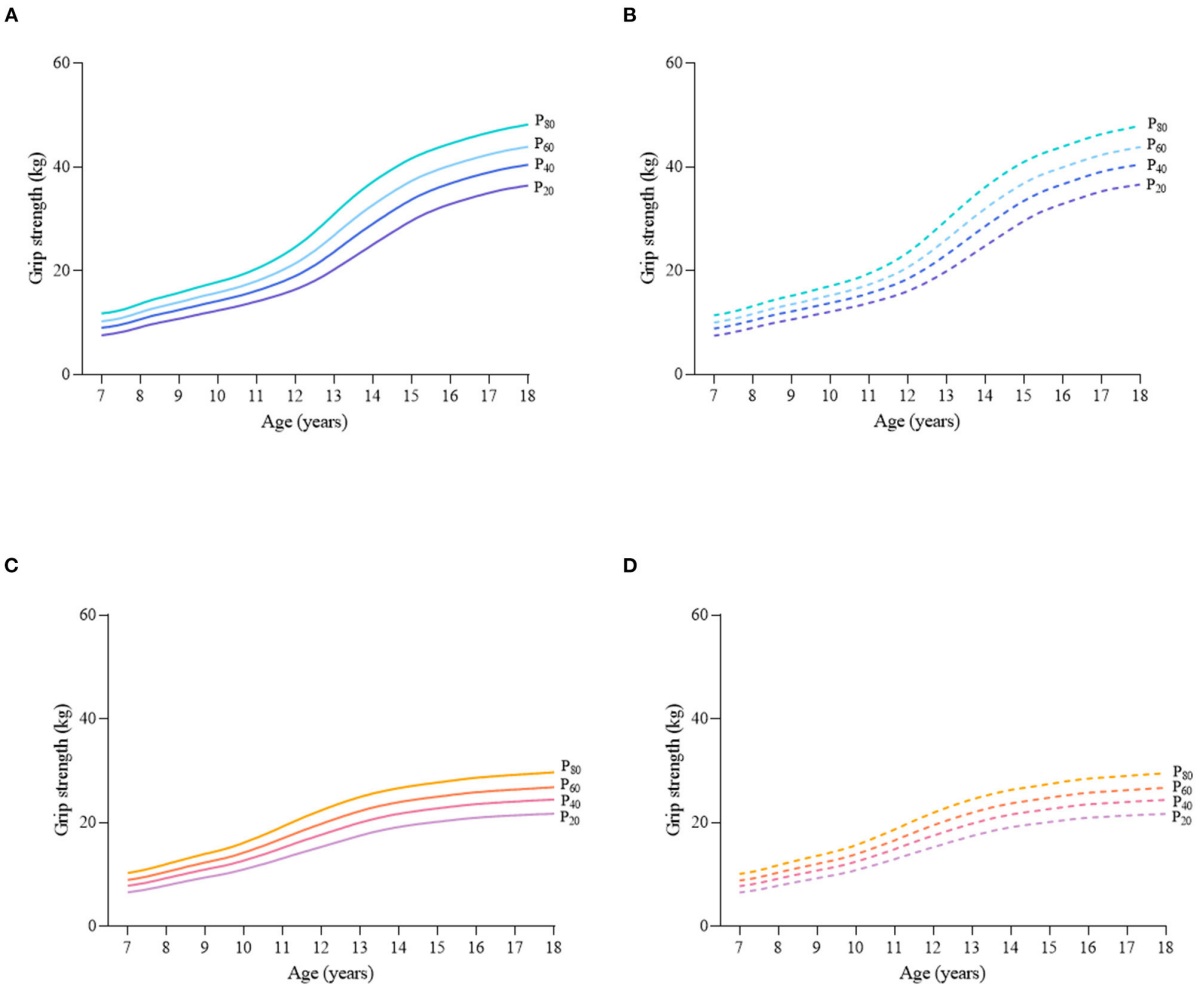
Sex- and age-specific percentile curves (P_20_, P_40_, P_60_, and P_80_) for grip strength (GS) (kg) for the Chinese children and adolescents aged 7–18 years based on total population and normal weight population. **(A)** Boys-Total population, **(B)** Boys-Normal weight population, **(C)** Girls-Total population, and **(D)** Girls-Normal weight population.

**Figure 7:**
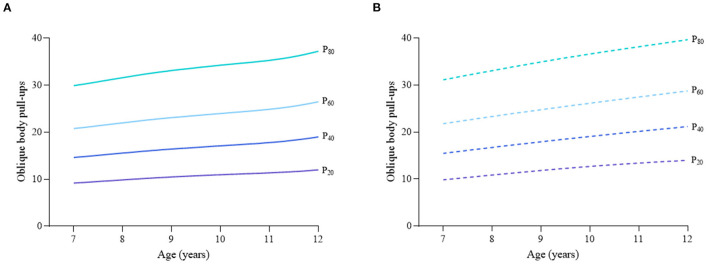
Age-specific percentile curves (P_20_, P_40_, P_60_, and P_80_) for oblique body pull-ups for the Chinese boys aged 7–12 years based on total population and normal weight population. **(A)** Boys-Total population and **(B)** Boys-Normal weight population.

**Figure 8:**
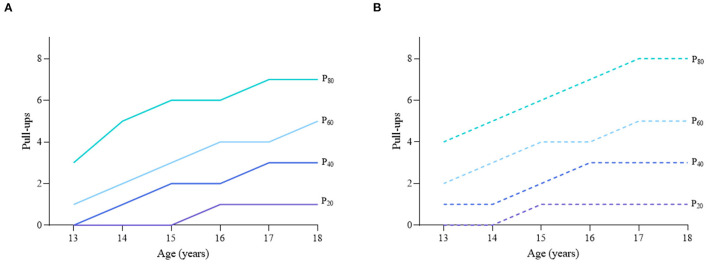
Age-specific percentile curves (P_20_, P_40_, P_60_, and P_80_) for pull-ups for the Chinese boys aged 13–18 years based on total population and normal weight population. **(A)** Boys-Total population and **(B)** Boys-Normal weight population.

**Figure 9:**
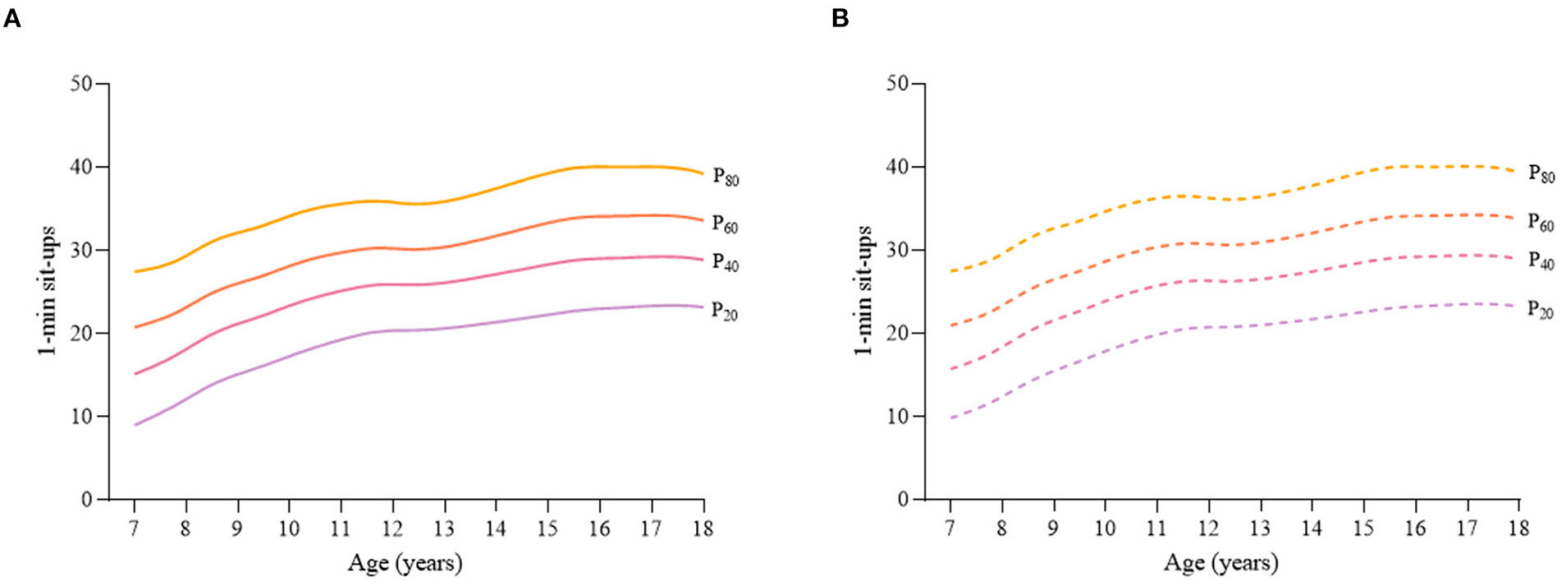
Age-specific percentile curves (P_20_, P_40_, P_60_, and P_80_) for 1-min sit-ups for the Chinese girls aged 7–18 years based on total population and normal weight population. **(A)** Girls-Total population and **(B)** Girls-Normal weight population.

**Figure 10:**
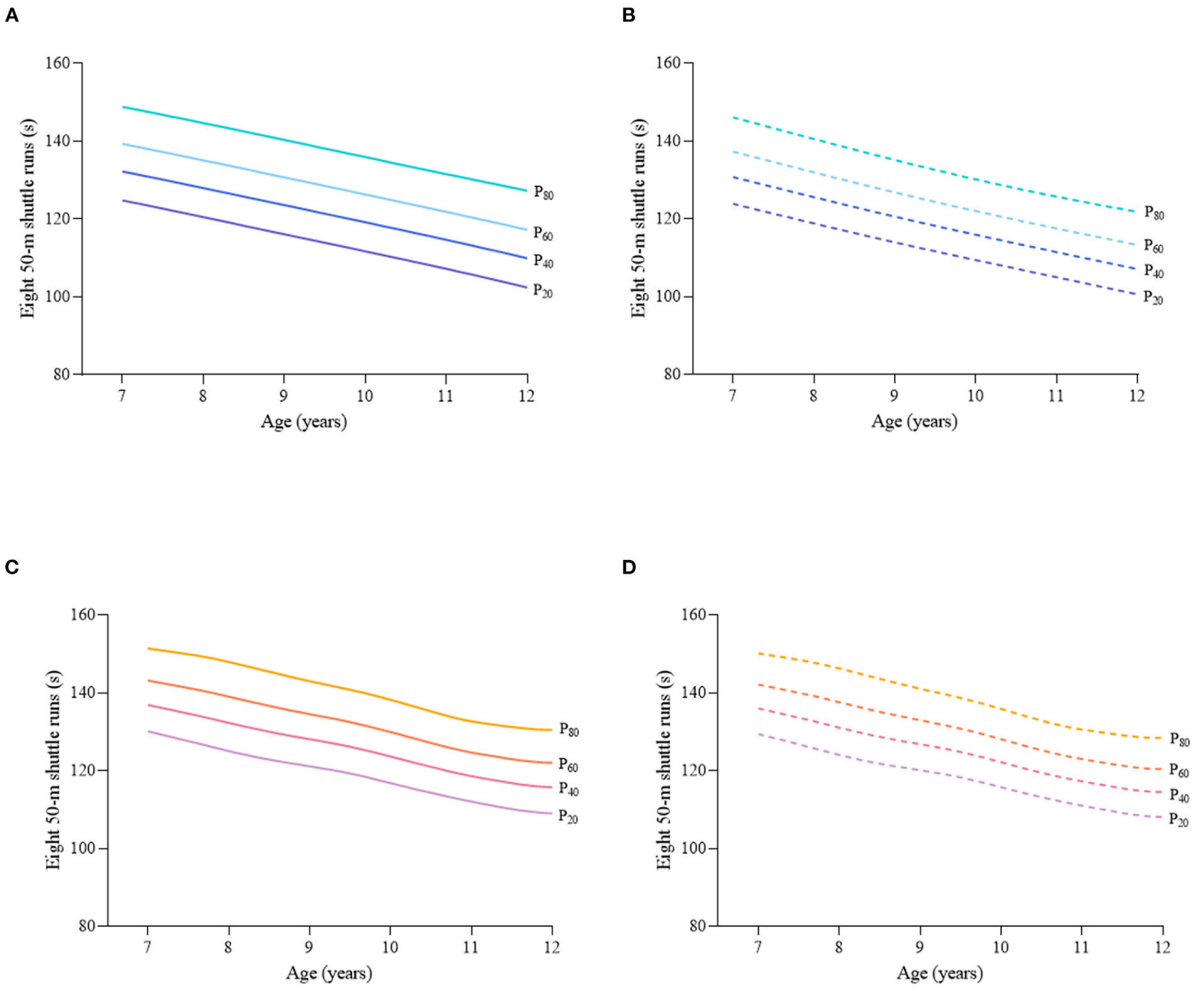
Sex- and age-specific percentile curves (P_20_, P_40_, P_60_, and P_80_) for eight 50-m shuttle runs (s) for the Chinese children and adolescents aged 7–12 years based on total population and normal weight population. **(A)** Boys-Total population, **(B)** Boys-Normal weight population, **(C)** Girls-Total population, and **(D)** Girls-Normal weight population.

**Figure 11:**
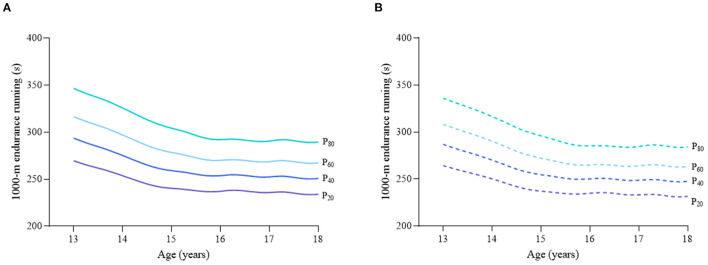
Age-specific percentile curves (P_20_, P_40_, P_60_, and P_80_) for 1,000-m endurance running (ER) for the Chinese boys aged 13–18 years based on total population and normal weight population. **(A)** Boys-Total population and **(B)** Boys-Normal weight population.

**Figure 12:**
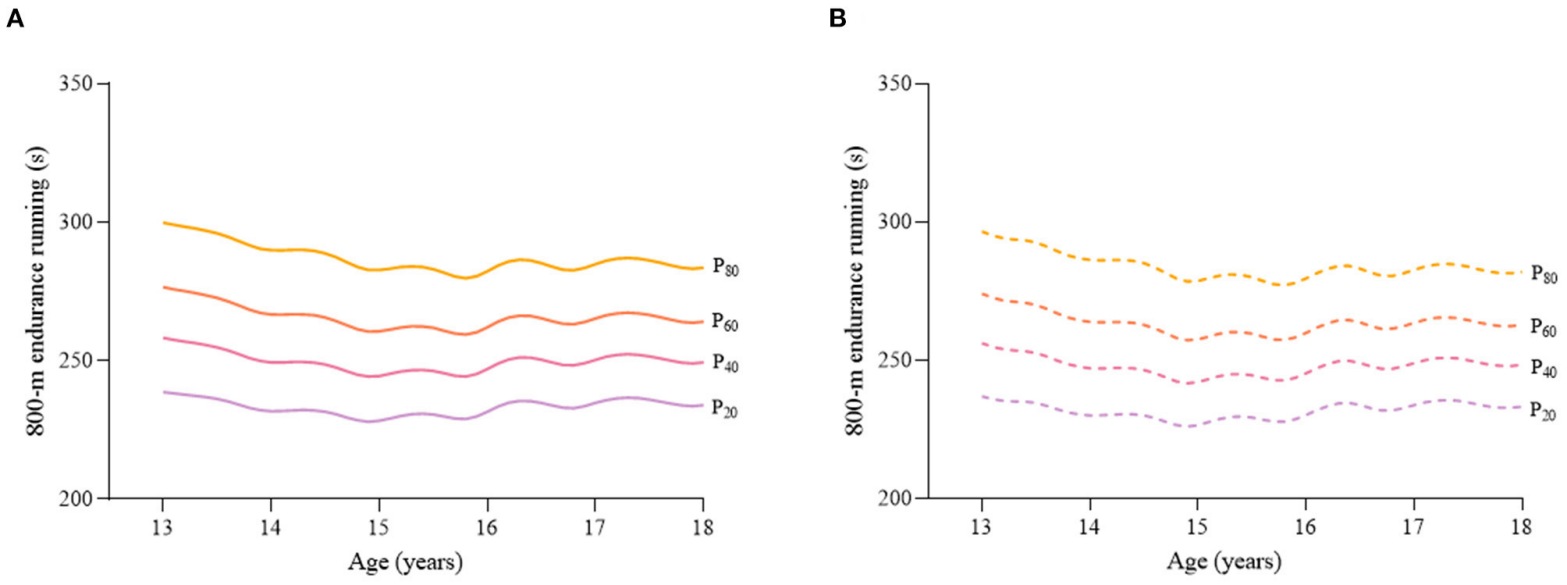
Age-specific percentile curves (P_20_, P_40_, P_60_, and P_80_) for 800-m endurance running (ER) (s) for the Chinese girls aged 13–18 years based on total population and normal weight population. **(A)** Girls-Total population and **(B)** Girls-Normal weight population.

Considering the different characteristics of physical fitness in each subgroup of sex, age group, and nutritional status among children and adolescents, Figures 2–12 and Supplementary Tables 2–15 present the sex- and age-specific percentile curves (P_20_, P_40_, P_60_, and P_80_) and percentile values (P_5_, P_10_, P_20_, P_30_, P_40_, P_50_, P_60_, P_70_, P_80_, P_90_, and P_95_) for the seven physical fitness components based on the total and the normal weight population, respectively. Irrespective of the total population or the normal weight population, for both sexes, their performances increased with age in all components except for SR, which revealed the worst performance at P_50_ among boys aged 12 years and girls aged 11 years. Almost all components, such as FVC, SLJ, 50-m dash, GS, 1-min sit-ups, and ER, increased with age at each percentile, while for pull-ups, the pronounced increases occurred only in higher percentiles (P_60_ and above). The annual increments of all components were larger for boys than girls at the peak time, which was earlier in girls than boys, i.e., for FVC at P_50_, 400.0 ml/year for boys at 14 years old compared with 211.8 ml/year for girls at 12 years old among the total population, and 409.9 ml/year for boys at 14 years old compared with 212.0 ml/year for girls at 11 years old among normal weight population; for SLJ at P_50_, 15.7 cm/year for boys at 13 years old compared with 9.8 cm/year for girls at 8 years old among the total population and 15.2 cm/year for boys at 13 and 14 years old compared with 10.3 cm/year for girls at 8 years old among normal weight population, respectively. The total increase of each component was also higher among boys than girls in FVC (2,693.0 ml for boys vs. 1,472.3 ml for girls among the total population and 2,695.8 ml for boys vs. 1,468.6 ml for girls among normal weight population), SLJ (108.4 cm for boys vs. 56.4 cm for girls among total population and 109.5 cm for boys vs. 56.3 cm for girls among normal weight population), 50-m dash (−3.76 s for boys vs. −2.19 s for girls among the total population and −3.71 s for boys vs. −2.15 s for girls among normal weight population), SR (4.80 cm for boys vs. 3.60 cm for girls among total population and 5.28 cm for boys vs. 3.56 cm for girls among normal weight population), GS (32.50 kg for boys vs. 17.25 kg for girls among the total population and 32.76 kg for boys vs. 17.26 kg for girls among normal weight population), and eight 50-m shuttle runs (−22.21 s for boys vs. −21.16 s for girls among the total population and −23.84 s for boys vs. −21.57 s for girls among normal weight population, Table 2). Further, the physical fitness performances stabilized earlier in girls than boys.

**Table 2:** Annual and total increments of physical fitness components at P_50_ among the total and normal weight population.

**Age (years)**	**7–8**	**8–9**	**9–10**	**10–11**	**11–12**	**12–13**	**13–14**	**14–15**	**15–16**	**16–17**	**17–18**	**Total**
**Total population**												
**Boys**												
Forced vital capacity (ml)	158.2	193.7	204.5	209.7	267.3	349.2	400.0	371.3	253.4	193.0	92.7	2,693.0
Standing long jump (cm)	10.4	9.2	7.7	7.7	9.4	15.7	15.2	14.5	10.8	5.4	2.4	108.4
50-m dash (s)	−0.65	−0.50	−0.34	−0.31	−0.33	−0.48	−0.48	−0.36	−0.17	−0.12	−0.02	−3.76
Sit-and-reach (cm)	−0.22	−0.39	−0.82	−0.40	−0.22	0.84	1.44	1.81	1.53	0.72	0.51	4.80
Grip strength (kg)	1.69	1.86	1.79	2.09	3.12	5.03	5.63	4.65	3.04	2.13	1.45	32.50
Oblique body pull-ups	1.1	0.9	0.8	0.8	1.4	–	–	–	–	–	–	5.0
Pull-Ups	–	–	–	–	–	–	1.0	0.0	1.0	0.0	1.0	3.0
Eight 50-m shuttle runs (s)	−4.24	−4.37	−4.40	−4.49	−4.71	–	–	–	–	–	–	−22.21
1,000-m endurance running (s)	–	–	–	–	–	–	−19.13	−17.27	−6.77	−1.38	−1.44	−45.99
**Girls**												
Forced vital capacity (ml)	136.0	160.6	189.2	210.8	211.8	182.5	138.2	99.4	88.4	56.7	−1.3	1,472.3
Standing long jump (cm)	9.8	8.8	7.8	7.9	5.9	5.2	3.3	3.6	4.1	0.2	−0.2	56.4
50-m dash (s)	−0.66	−0.50	−0.37	−0.33	−0.22	−0.12	−0.07	−0.02	0.02	0.02	0.06	−2.19
Sit-and-reach (cm)	0.11	−0.36	−0.39	−0.08	0.09	0.60	1.15	0.98	0.82	0.41	0.27	3.60
Grip strength (kg)	1.51	1.73	1.82	2.58	2.67	2.40	1.70	1.06	0.83	0.51	0.43	17.25
1-min sit-ups	2.7	3.0	2.1	1.7	0.7	0.2	1.2	1.3	0.8	0.1	−0.5	13.3
Eight 50-m shuttle runs (s)	−4.40	−4.33	−4.54	−5.13	−2.76	–	–	–	–	–	–	−21.16
800-m endurance running (s)	–	–	–	–	–	–	−9.43	−5.51	2.01	3.28	−1.08	−10.73
**Normal weight population**												
**Boys**												
Forced vital capacity (ml)	153.1	177.2	191.0	212.7	256.1	341.6	409.9	388.8	268.0	200.5	96.9	2,695.8
Standing long jump (cm)	11.3	10.1	8.6	7.5	9.4	15.2	15.2	14.0	10.5	5.4	2.3	109.5
50-m dash (s)	−0.65	−0.53	−0.37	−0.33	−0.31	−0.44	−0.45	−0.33	−0.17	−0.11	−0.02	−3.71
Sit-and-reach (cm)	−0.11	−0.35	−0.62	−0.38	−0.25	0.67	1.54	1.83	1.55	0.77	0.63	5.28
Grip strength (kg)	1.63	1.80	1.67	1.98	3.07	5.01	5.71	4.90	3.14	2.40	1.46	32.76
Oblique body pull-ups	1.4	1.3	1.3	1.2	1.2	–	–	–	–	–	–	6.4
Pull-ups	–	–	–	–	–	–	1.0	1.0	0.0	1.0	0.0	3.0
Eight 50-m shuttle runs (s)	−5.24	−5.05	−4.74	−4.46	−4.34	–	–	–	–	–	–	−23.83
1,000-m endurance running (s)	–	–	–	–	–	–	−17.41	−16.69	−5.77	−1.23	−0.94	−42.04
**Girls**												
Forced vital capacity (ml)	133.1	150.3	183.5	212.0	209.3	186.7	145.4	99.6	98.2	48.4	2.1	1,468.6
Standing long jump (cm)	10.3	9.0	8.1	7.9	5.9	4.8	3.1	3.5	3.9	0.1	−0.3	56.3
50-m dash (s)	−0.65	−0.51	−0.38	−0.32	−0.22	−0.11	−0.06	−0.02	0.03	0.03	0.06	−2.15
Sit-and-reach (cm)	0.04	−0.39	−0.39	−0.15	0.02	0.68	1.29	1.07	0.82	0.37	0.2	3.56
Grip strength (kg)	1.49	1.61	1.78	2.56	2.76	2.37	1.76	1.09	0.92	0.48	0.44	17.26
1-min sit-ups	2.6	3.1	2.3	1.8	0.4	0.2	1.1	1.2	0.7	0.1	−0.5	13.0
Eight 50-m shuttle runs (s)	−4.73	−4.44	−4.74	−5.01	−2.65	–	–	–	–	–	–	−21.57
800-m endurance running (s)	–	–	–	–	–	–	−9.68	−5.68	2.82	3.65	−0.62	−9.51

**Figure 13:**
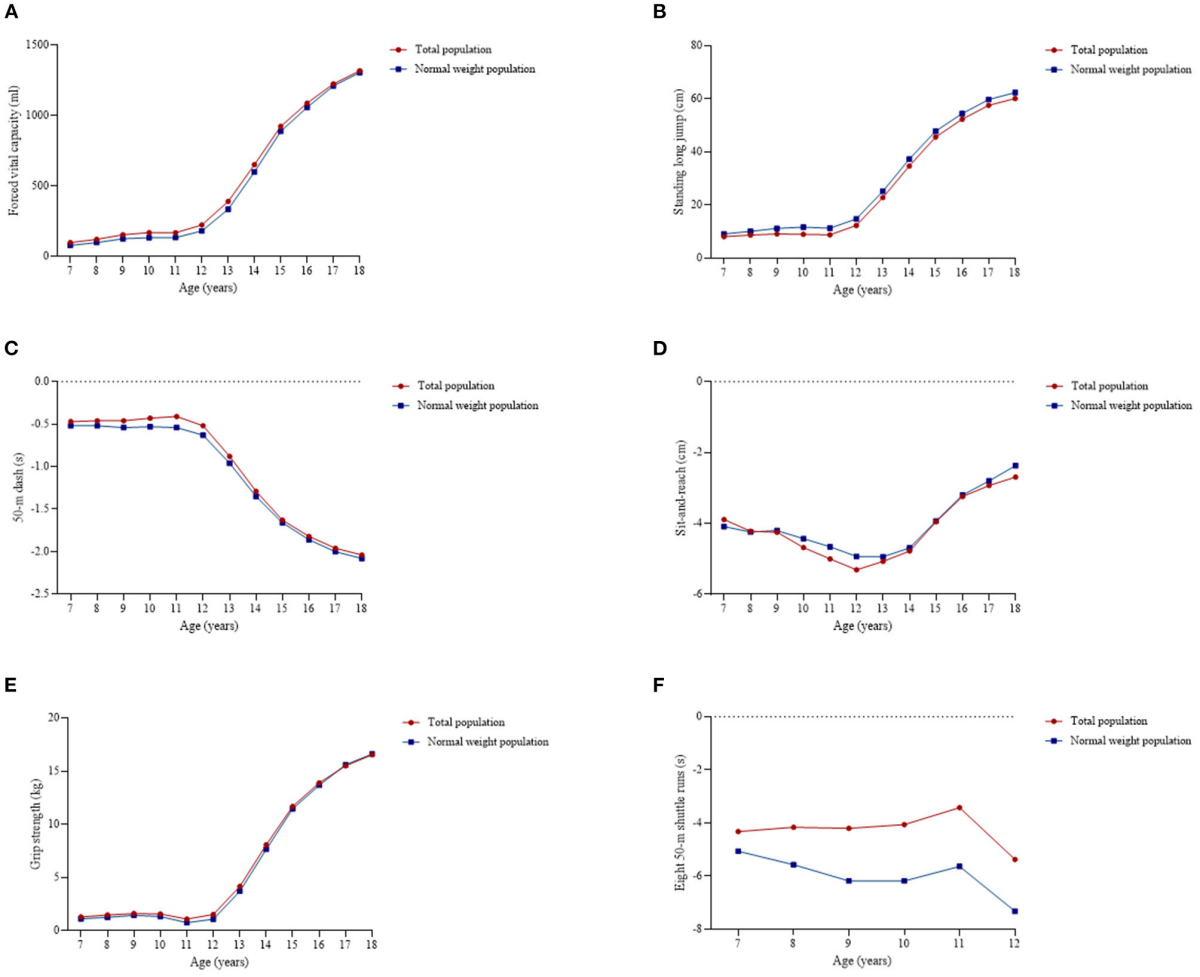
Sex differences of the physical fitness components at P_50_ among total and normal weight population. **(A)** Forced vital capacity, **(B)** Standing long jump, **(C)** 50-m dash, **(D)** Sit-and-reach, **(E)** Grip strength, and **(F)** Eight 50-m shuttle runs.

For both total and normal weight population, the sex differences of each component remained small and stable before the age of 12, then increased with age in FVC (the differences were 155.8 ml for average before 12 years old vs. 933.4 ml for average after 12 years old among total population and 125.1 ml for average before 12 years old vs. 900.1 ml for average after 12 years old among normal weight population at P_50_), SLJ (the differences were 9.3 cm for average before 12 years old vs. 45.5 cm for average after 12 years old among total population and 11.4 cm for average before 12 years old vs. 47.8 cm for average after 12 years old among normal weight population at P_50_), GS (the differences were 1.42 kg for average before 12 years old vs. 11.64 kg for average after 12 years old among total population and 1.16 kg for average before 12 years old vs. 11.45 kg for average after 12 years old among normal weight population at P_50_), and 50-m dash (the differences were 0.46 s for average before 12 years old vs. 1.60 s for average after 12 years old among total population and 0.54 s for average before 12 years old vs. 1.65 s for average after 12 years old among normal weight population at P_50_). As for SR, it displayed the greatest sex difference at the age of 12 (Figure 13).

## Discussion

This study presents age- and sex-specific percentile curves for seven components of physical fitness (FVC, SLJ, 50-m dash, SR, GS, BMS, and ER) among the total and normal weight population based on a large nationally representative sample of Chinese Han children and adolescents. To the best of our knowledge, it is the first study to generate percentile values for the physical fitness components based on both the total and the normal weight population at the national level with a representative sample, which are helpful as a reference for regional comparisons and for monitoring the trends of physical fitness over time.

Given that physical fitness strongly indicates current and future health outcomes for children and adolescents, it is important to assess the relative position and identify low levels of physical fitness (30). For instance, children and adolescents below the 5th percentile should be considered a population with a “warning signal” and would be recommended to undergo clinical evaluation for the presence of comorbidities (31). Furthermore, from the perspective of prevention, these percentile values could help identify the physical fitness components in need of improvement. Some studies have suggested using the normative quintile-based framework to classify the physical fitness levels, where those below the 20th percentile are classified as “very low/poor,” 20th−40th percentiles as “low/poor,” 40th−60th percentiles as “moderate,” 60th−80th percentiles as “high/good,” and those above the 80th percentile as “very high/good”(16, 32–34). Moreover, for the individuals with “very low” or “low” level of physical fitness who were prescribed intervention, by tracking their percentile categories, the precision and influence of the intervention could be assessed (33). Additionally, tracking these quintile-based thresholds over a period of time, the secular trends for each physical fitness component might be monitored (16), and for those components with poor performance, the policymakers should consider specific interventions and efforts to improve the status of the entire population. For example, a great quantity of 0 scores of pull-ups test reflected the lack of BMS training, and the policymakers and school health providers might need to consider re-designing the physical education curriculum and adding some courses related to these core items of physical fitness. Moreover, a supportive environment for physical activity and targeted training, such as professional physical education teachers, qualified sports ground, and equipment were also needed.

Contrarily, the high percentile values could also contribute additional, i.e., they could be used by physical education teachers or coaches for sports talent identification. Some studies have suggested that children and adolescents with a score above the 95th percentile might be considered a talent for relevant sports (35) and could be recruited into some sporting or athletic programs. For instance, soccer players demonstrated superior performance in sports-specific speed and power in the lower limbs (36). Power, speed, isometric and explosive strength, strength endurance, and dynamic and static flexibility are the most determinant physical fitness aspects of the talent selection process in artistic gymnasts (37).

Our study found that boys performed better than girls in FVC, SLJ, 50-m dash, GS, and ER, but worse in SR. Meanwhile, almost all performances in physical fitness components increased with age, although they appeared to plateau at a certain age. These findings are consistent with the results of previous studies from various countries (3, 27, 28, 30, 38). Sex difference might be due to some biological factors, such as fat mass and fat-free mass (17, 39); the rapid increase in boys and mild increase in girls of fat-free mass after puberty might lead to the differences in muscle strength (12), the lower tissue density in girls due to the higher percentage of fat mass and a lower percentage of fat-free mass among them might explain their better flexibility (17), and the differences in ER might be explained by differences in mechanical efficiency and/or the fractional utilization of oxygen (16). Meanwhile, some environmental factors might also lead to the sex difference, such as social interests, peer influence, or lack of motivation toward physical activity, causing girls to be less active than boys (40). The age-related differences are generally attributed to the distinct development, growth, and maturation during childhood and adolescence (41). Moreover, these fitness components are dependent on the development of experience and skills over time (42).

However, the age trend of SR showed different results across studies. We found that both boys and girls had the worst SR performance at the age of 11 or 12 years. Iglesias-Soler et al. (43) had similar findings to ours, but Zhang et al. (18) found that the performance of SR increased with age. In our study, children and adolescents received a positive score when their fingertips exceeded the toes. During early puberty, the growth of the legs is faster than that of the arms (44), which might explain the poor performance during this period.

We observed that a substantial number of boys scored 0 in the pull-ups test, such as 40% boys aged 13 years and 30% boys aged 14 years, suggesting that this test might not finely distinguish the arm muscle strength of boys. Therefore, its usefulness and future use in Chinese children and adolescents are questionable due to the lack of sensitivity in this test, and more accurate alternative tests, such as bent-arm hang/flexed-arm hang, are widely used in some countries and regions (16, 35, 40, 43, 45, 46) might also be considered for use. Moreover, studies exploring the relationship between the performances of pull-ups and bent-arm hang/flexed-arm hang are needed in the future to fully use previous data. Meanwhile, GS was an important indicator of muscular endurance and overall strength (47), its combination with BMS could complement each other. In this study, we found that the GS performance of total population was slightly higher than that of normal weight population, as GS was considered to be affected by weight (48). In such cases, the percentile values based on the normal weight population might alleviate the effect of weight.

Another study conducted in the Chinese children and adolescents established percentile values for physical fitness, and two components, SLJ and 50-m dash, were measured similar to ours, and so were the findings (18). Compared with other countries, taking sex- and age-specific P_50_ as an example, we found that the Chinese children and adolescents had better performance of SLJ than the European (3, 33), Columbian (49), Greek, and Macedonian children and adolescents (35). However, regarding 50-m dash, children and adolescents from many countries, such as France (28), Germany (17), Australia (34), and Korea (12) performed better than Chinese children and adolescents. Meanwhile, the Chinese children and adolescents performed better than Polish in SR, but worse in ER (40). Overall, the construction of these percentile curves might help to locate the performance of each physical fitness component of Chinese children and adolescents around the world and focus on the priorities for intervention; for example, in comparison with other countries, the weaknesses of physical fitness among Chinese children and adolescents might be the speed quality and endurance quality; therefore, targeted measures to enhance these two physical fitness components are urgent. Furthermore, other countries with better performance might provide advanced experiences for enhancing the physical fitness of children and adolescents. All of these benefits could help the Chinese government to develop a short- to long-term strategic planning regarding the physical fitness and health of children and adolescents.

This study has several strengths, including the use of a large nationally representative sample, the use of objective measures of physical fitness was conducted by trained staff and standardized procedures, and the use of GAMLSS that expands on the LMS technique. Despite these strengths, this study reveals some limitations, i.e., the cross-sectional design. Taking the changes of individual growth and maturation in children and adolescents into account, physical fitness normative values should be obtained from longitudinal studies. Nevertheless, cross-sectional data collected by harmonized and standardized procedures, and analyzed by appropriate statistical methods in the absence of longitudinal studies are also valuable.

## Conclusions

Physical fitness in childhood and adolescence is a powerful marker of health. This study established sex- and age-specific percentile values of seven physical fitness components for the Chinese children and adolescents aged 7–18 years based on the total and the normal weight population. Boys performed better than girls in FVC, SLJ, 50-m dash, GS, and ER, but worse in SR. The performances of FVC, SLJ, 50-m dash, GS, BMS, and ER increased with age, but the estimates of SR were at the bottom among boys aged 12 years and girls aged 11 years. These percentile values might help to identify children and adolescents with poor physical fitness to give appropriate interventions and monitor longitudinal changes and help to recruit some talented children and adolescents with good physical fitness into athletic development programs.

## Data Availability Statement

The data analyzed in this study is subject to the following licenses/restrictions: All the individual (de-identified) participant data collected in the surveys can be shared with investigators whose proposed use of the data has been approved by an independent review committee identified for this purpose by contacting the corresponding author. Requests to access these datasets should be directed to majunt@bjmu.edu.cn and songyi@bjmu.edu.cn.

## Ethics Statement

The studies involving human participants were reviewed and approved by Medical Research Ethics Committee of the Peking University Health Science Center. Written informed consent to participate in this study was provided by the participants' legal guardian/next of kin.

## Author Contributions

NM conceptualized and designed the study, completed the statistical analyses, drafted the initial manuscript, and reviewed and revised the manuscript. YD and YS contributed to the conceptualization and design of the study, supervised the data collection, the statistical analyses and initial drafting of the manuscript, and reviewed and revised the manuscript. PL participated in conceiving the study design and critically reviewed and revised the manuscript from preliminary draft to submission. JD, YL, PZ, XY, JZ, and JM assisted with the data interpretation and reviewed and revised the manuscript. All authors approved the final manuscript as submitted and agreed to be accountable for all aspects of the work.

## Funding

This study was supported by the Humanities and Social Sciences Planning Fund Project, Ministry of Education, People's Republic of China (Grant No. 19YJA890022 to YS), and National Statistical Science Research Project (2021LY052 to YS).

## Conflict of Interest

The authors declare that the research was conducted in the absence of any commercial or financial relationships that could be construed as a potential conflict of interest.

## Publisher's Note

All claims expressed in this article are solely those of the authors and do not necessarily represent those of their affiliated organizations, or those of the publisher, the editors and the reviewers. Any product that may be evaluated in this article, or claim that may be made by its manufacturer, is not guaranteed or endorsed by the publisher.
