# Supplementary material for: Percentile Curves for Multiple Physical Fitness Components Among Chinese Han Children and Adolescents Aged 7–18 Years From a National Survey Based on the Total and the Normal Weight Population
Source: Front Nutr. 2022 Jan 3;8:770349. doi: 10.3389/fnut.2021.770349 (PMC8762235; doi:10.3389/fnut.2021.770349)
Supplement: Supplementary file 1 [file Data_Sheet_1.docx]

Supplementary Material

# Supplementary Figures and Tables

## Supplementary Figures

**Figure legends**

**Figure S1.** Testing method of Sit-and-reach

**Figure S2.** Testing method of Oblique body pull-ups

**Figure S3.** Testing method of Pull-ups

**Figure S4.** Worm plots and Q-Q plots of selected models for Forced vital capacity among total population and normal weight population

**Figure S5.** Worm plots and Q-Q plots of selected models for Standing long jump among total population and normal weight population

**Figure S6.** Worm plots and Q-Q plots of selected models for 50-m dash among total population and normal weight population

**Figure S7.** Worm plots and Q-Q plots of selected models for Sit-and-reach among total population and normal weight population

**Figure S8.** Worm plots and Q-Q plots of selected models for Grip strength among total population and normal weight population

**Figure S9.** Worm plots and Q-Q plots of selected models for Oblique body pull-ups among total population and normal weight population

**Figure S10.** Worm plots and Q-Q plots of selected models for 1-min sit-ups among total population and normal weight population

**Figure S11.** Worm plots and Q-Q plots of selected models for Eight 50-m shuttle runs among total population and normal weight population

**Figure S12.** Worm plots and Q-Q plots of selected models for 800-/1000-m endurance running among total population and normal weight population

**Figure S1** Testing method of Sit-and-reach


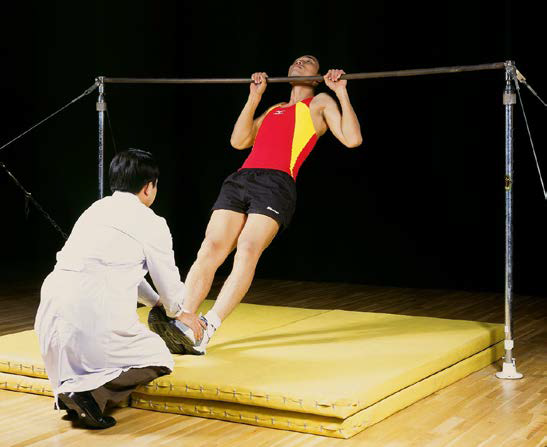


**Figure S2** Testing method of Oblique body pull-ups


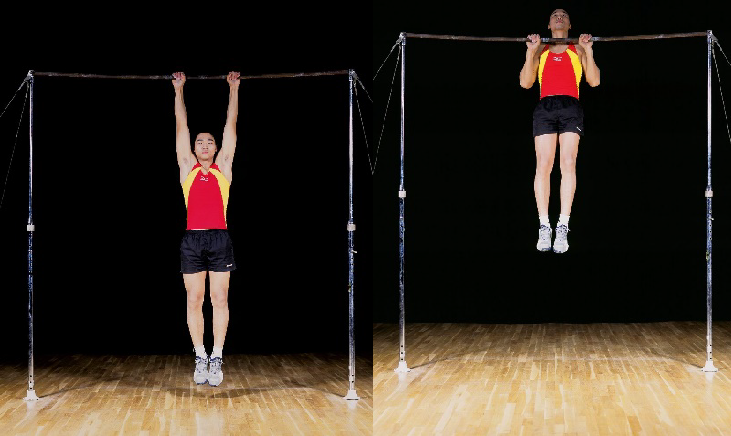


**Figure S3** Testing method of Pull-ups

**Figure S4** Worm plots and Q-Q plots of selected models for Forced vital capacity among total population and normal weight population

**Figure S5** Worm plots and Q-Q plots of selected models for Standing long jump among total population and normal weight population

**Figure S6** Worm plots and Q-Q plots of selected models for 50-m dash among total population and normal weight population

**Figure S7** Worm plots and Q-Q plots of selected models for Sit-and-reach among total population and normal weight population

**Figure S8** Worm plots and Q-Q plots of selected models for Grip strength among total population and normal weight population

**Figure S9** Worm plots and Q-Q plots of selected models for Oblique body pull-ups among total population and normal weight population

**Figure S10** Worm plots and Q-Q plots of selected models for 1-min sit-ups among total population and normal weight population

**Figure S11** Worm plots and Q-Q plots of selected models for Eight 50-m shuttle runs among total population and normal weight population

**Figure S12** Worm plots and Q-Q plots of selected models for 800-/1000-m endurance running among total population and normal weight population

## Supplementary Tables

**Table S1** Nutritional status of Chinese children and adolescents aged 7–18 years in 2014 CNSSCH

|  | | Normal weight | |  | Thinness | |  | Overweight ^a^ | | |
| --- | --- | --- | --- | --- | --- | --- | --- | --- | --- | --- |
|  |  | N | % |  | N | % |  | | N | % |
| Sex | Boys | 74453 | 69.5 |  | 4680 | 4.4 |  | 28006 | | 26.1 |
|  | Girls | 87546 | 81.8 |  | 3827 | 3.6 |  | 15668 | | 14.6 |
| Age(years) | 7 | 13388 | 75.0 |  | 564 | 3.2 |  | 3906 | | 21.9 |
|  | 8 | 12848 | 72.0 |  | 606 | 3.4 |  | 4396 | | 24.6 |
|  | 9 | 12367 | 69.0 |  | 664 | 3.7 |  | 4895 | | 27.3 |
|  | 10 | 12026 | 67.0 |  | 789 | 4.4 |  | 5125 | | 28.6 |
|  | 11 | 12126 | 67.7 |  | 753 | 4.2 |  | 5038 | | 28.1 |
|  | 12 | 12715 | 71.0 |  | 772 | 4.3 |  | 4409 | | 24.6 |
|  | 13 | 13450 | 74.9 |  | 691 | 3.9 |  | 3806 | | 21.2 |
|  | 14 | 13998 | 78.1 |  | 673 | 3.8 |  | 3257 | | 18.2 |
|  | 15 | 14570 | 81.2 |  | 701 | 3.9 |  | 2679 | | 14.9 |
|  | 16 | 14919 | 83.2 |  | 689 | 3.8 |  | 2315 | | 12.9 |
|  | 17 | 15025 | 83.8 |  | 777 | 4.3 |  | 2138 | | 11.9 |
|  | 18 | 14567 | 85.2 |  | 828 | 4.8 |  | 1710 | | 10.0 |
| Total | | 161999 | 75.6 |  | 8507 | 4.0 |  | 43674 | | 20.4 |

Note: ^a^ The children and adolescents with obesity were included.

**Table S2** Forced vital capacity (ml) percentile values by age group and sex among Chinese children and adolescents aged 7–18 years based on total population

| Age（years） | P_5_ | P_10_ | P_20_ | P_30_ | P_40_ | P_50_ | P_60_ | P_70_ | P_80_ | P_90_ | P_95_ |
| --- | --- | --- | --- | --- | --- | --- | --- | --- | --- | --- | --- |
| Boys |  |  |  |  |  |  |  |  |  |  |  |
| 7 | 576.4 | 662.2 | 778.5 | 872.0 | 958.9 | 1045.8 | 1136.4 | 1235.1 | 1351.8 | 1515.1 | 1651.3 |
| 8 | 663.6 | 764.7 | 900.0 | 1007.4 | 1106.1 | 1204.0 | 1305.8 | 1416.9 | 1549.0 | 1735.3 | 1891.7 |
| 9 | 783.3 | 901.1 | 1056.8 | 1178.5 | 1289.0 | 1397.7 | 1510.2 | 1633.4 | 1780.4 | 1989.1 | 2165.5 |
| 10 | 921.0 | 1054.9 | 1229.4 | 1363.8 | 1484.6 | 1602.2 | 1723.6 | 1856.8 | 2016.3 | 2244.0 | 2437.5 |
| 11 | 1033.4 | 1188.8 | 1389.6 | 1542.9 | 1679.6 | 1811.9 | 1948.3 | 2098.3 | 2279.0 | 2538.3 | 2760.2 |
| 12 | 1188.7 | 1368.9 | 1599.9 | 1774.9 | 1929.9 | 2079.2 | 2232.9 | 2402.3 | 2606.9 | 2902.0 | 3155.4 |
| 13 | 1390.6 | 1602.8 | 1873.2 | 2076.8 | 2256.3 | 2428.4 | 2605.4 | 2800.6 | 3037.0 | 3378.9 | 3673.3 |
| 14 | 1647.3 | 1891.5 | 2200.8 | 2432.1 | 2634.8 | 2828.4 | 3027.1 | 3246.4 | 3512.2 | 3897.1 | 4228.9 |
| 15 | 1942.2 | 2206.0 | 2537.1 | 2782.6 | 2996.5 | 3199.7 | 3407.6 | 3637.1 | 3915.3 | 4318.3 | 4665.9 |
| 16 | 2208.0 | 2473.3 | 2803.0 | 3045.3 | 3254.9 | 3453.1 | 3655.3 | 3878.4 | 4149.1 | 4541.4 | 4880.0 |
| 17 | 2415.7 | 2680.9 | 3008.2 | 3247.1 | 3452.7 | 3646.1 | 3843.1 | 4060.7 | 4325.1 | 4709.1 | 5041.0 |
| 18 | 2482.7 | 2755.3 | 3090.4 | 3334.0 | 3542.9 | 3738.8 | 3938.3 | 4159.2 | 4428.4 | 4820.9 | 5161.3 |
| Girls |  |  |  |  |  |  |  |  |  |  |  |
| 7 | 508.8 | 589.8 | 699.0 | 786.3 | 866.9 | 947.1 | 1030.6 | 1121.5 | 1229.1 | 1379.9 | 1505.8 |
| 8 | 598.1 | 688.4 | 809.7 | 906.1 | 994.9 | 1083.1 | 1174.9 | 1275.0 | 1393.7 | 1560.7 | 1700.8 |
| 9 | 703.0 | 804.7 | 940.4 | 1047.8 | 1146.3 | 1243.7 | 1345.0 | 1455.6 | 1587.0 | 1772.4 | 1928.2 |
| 10 | 811.0 | 929.1 | 1085.9 | 1209.2 | 1321.8 | 1432.9 | 1548.1 | 1674.0 | 1823.7 | 2035.4 | 2213.7 |
| 11 | 919.2 | 1058.1 | 1241.6 | 1385.1 | 1515.5 | 1643.7 | 1776.4 | 1921.4 | 2094.1 | 2338.6 | 2544.6 |
| 12 | 1050.0 | 1206.6 | 1411.9 | 1571.1 | 1714.9 | 1855.5 | 2000.7 | 2159.1 | 2347.8 | 2614.8 | 2840.0 |
| 13 | 1189.7 | 1357.4 | 1574.9 | 1742.2 | 1892.1 | 2038.0 | 2188.0 | 2351.5 | 2546.3 | 2821.8 | 3054.2 |
| 14 | 1294.4 | 1471.0 | 1698.4 | 1871.9 | 2026.6 | 2176.2 | 2329.8 | 2497.2 | 2696.6 | 2979.1 | 3217.4 |
| 15 | 1370.4 | 1553.7 | 1788.2 | 1966.0 | 2123.7 | 2275.6 | 2431.4 | 2601.2 | 2803.8 | 3091.3 | 3334.4 |
| 16 | 1469.3 | 1652.5 | 1885.4 | 2060.8 | 2215.5 | 2364.0 | 2516.0 | 2681.9 | 2880.0 | 3161.9 | 3400.7 |
| 17 | 1548.1 | 1728.2 | 1956.1 | 2126.9 | 2277.0 | 2420.7 | 2567.6 | 2728.4 | 2921.0 | 3196.0 | 3429.8 |
| 18 | 1550.8 | 1730.4 | 1957.4 | 2127.4 | 2276.6 | 2419.4 | 2565.7 | 2726.3 | 2919.8 | 3197.9 | 3435.8 |

**Table S3** Forced vital capacity (ml) percentile values by age group and sex among Chinese children and adolescents aged 7–18 years based on normal weight population

| Age（years） | P5 | P10 | P20 | P30 | P40 | P50 | P60 | P70 | P80 | P90 | P95 |
| --- | --- | --- | --- | --- | --- | --- | --- | --- | --- | --- | --- |
| Boys |  |  |  |  |  |  |  |  |  |  |  |
| 7 | 566.4 | 646.7 | 757.0 | 846.9 | 931.4 | 1016.4 | 1105.2 | 1201.2 | 1313.4 | 1467.8 | 1594.4 |
| 8 | 653.1 | 749.2 | 878.2 | 980.8 | 1075.5 | 1169.5 | 1267.1 | 1373.2 | 1498.4 | 1673.4 | 1819.1 |
| 9 | 760.8 | 874.4 | 1023.5 | 1139.4 | 1244.1 | 1346.7 | 1452.7 | 1568.6 | 1706.8 | 1902.7 | 2068.2 |
| 10 | 890.2 | 1020.3 | 1187.8 | 1315.1 | 1428.3 | 1537.7 | 1650.2 | 1773.7 | 1922.3 | 2135.4 | 2317.4 |
| 11 | 1012.7 | 1164.3 | 1356.9 | 1501.6 | 1628.7 | 1750.4 | 1875.4 | 2013.3 | 2180.3 | 2421.9 | 2630.1 |
| 12 | 1162.9 | 1338.0 | 1559.3 | 1724.4 | 1868.8 | 2006.5 | 2147.8 | 2304.0 | 2493.7 | 2769.2 | 3007.3 |
| 13 | 1376.8 | 1579.7 | 1835.0 | 2024.9 | 2190.5 | 2348.1 | 2509.6 | 2687.8 | 2904.0 | 3217.5 | 3487.9 |
| 14 | 1637.3 | 1873.4 | 2169.0 | 2387.6 | 2577.7 | 2758.0 | 2942.2 | 3145.1 | 3390.7 | 3745.6 | 4050.9 |
| 15 | 1930.7 | 2191.1 | 2513.8 | 2750.3 | 2954.4 | 3146.8 | 3342.8 | 3558.9 | 3820.5 | 4199.1 | 4525.0 |
| 16 | 2201.3 | 2465.7 | 2790.0 | 3025.1 | 3226.3 | 3414.8 | 3606.5 | 3818.3 | 4075.8 | 4450.4 | 4774.4 |
| 17 | 2413.5 | 2678.0 | 3000.4 | 3232.8 | 3430.7 | 3615.3 | 3802.9 | 4011.0 | 4265.2 | 4636.8 | 4960.0 |
| 18 | 2487.6 | 2757.7 | 3086.6 | 3323.4 | 3524.7 | 3712.2 | 3902.9 | 4115.4 | 4376.1 | 4759.4 | 5094.6 |
| Girls |  |  |  |  |  |  |  |  |  |  |  |
| 7 | 506.9 | 587.4 | 695.2 | 780.8 | 859.6 | 937.7 | 1018.7 | 1106.8 | 1210.8 | 1356.4 | 1477.7 |
| 8 | 593.6 | 683.6 | 803.6 | 898.4 | 985.1 | 1070.8 | 1159.6 | 1256.1 | 1370.2 | 1529.9 | 1663.2 |
| 9 | 692.5 | 793.4 | 926.9 | 1031.7 | 1127.1 | 1221.1 | 1318.2 | 1423.8 | 1548.7 | 1723.8 | 1870.2 |
| 10 | 799.1 | 915.7 | 1069.2 | 1189.1 | 1297.8 | 1404.6 | 1514.8 | 1634.6 | 1776.6 | 1976.1 | 2143.1 |
| 11 | 910.2 | 1047.3 | 1227.1 | 1366.8 | 1493.0 | 1616.6 | 1744.0 | 1882.7 | 2047.2 | 2279.0 | 2473.6 |
| 12 | 1040.4 | 1194.7 | 1395.8 | 1550.8 | 1690.2 | 1825.9 | 1965.6 | 2117.6 | 2298.2 | 2552.7 | 2766.7 |
| 13 | 1182.2 | 1347.9 | 1561.7 | 1725.2 | 1871.1 | 2012.6 | 2157.7 | 2315.6 | 2503.2 | 2768.0 | 2990.7 |
| 14 | 1289.2 | 1464.6 | 1689.4 | 1860.1 | 2011.7 | 2158.0 | 2307.8 | 2470.8 | 2664.9 | 2939.5 | 3170.9 |
| 15 | 1366.3 | 1548.0 | 1779.7 | 1954.5 | 2109.0 | 2257.6 | 2409.6 | 2575.4 | 2773.2 | 3053.9 | 3291.3 |
| 16 | 1471.4 | 1653.7 | 1884.5 | 2057.6 | 2209.9 | 2355.8 | 2504.9 | 2667.8 | 2862.7 | 3140.4 | 3376.2 |
| 17 | 1543.3 | 1722.2 | 1947.6 | 2115.9 | 2263.4 | 2404.2 | 2548.1 | 2705.9 | 2895.4 | 3166.9 | 3398.6 |
| 18 | 1549.3 | 1727.8 | 1952.6 | 2120.0 | 2266.5 | 2406.3 | 2549.3 | 2706.9 | 2897.3 | 3172.3 | 3408.8 |

**Table S4** Standing long jump (cm) percentile values by age group and sex among Chinese children and adolescents aged 7–18 years based on total population

| Age（years） | P_5_ | P_10_ | P_20_ | P_30_ | P_40_ | P_50_ | P_60_ | P_70_ | P_80_ | P_90_ | P_95_ |
| --- | --- | --- | --- | --- | --- | --- | --- | --- | --- | --- | --- |
| Boys |  |  |  |  |  |  |  |  |  |  |  |
| 7 | 87.7 | 94.7 | 102.9 | 108.7 | 113.6 | 118.0 | 122.5 | 127.2 | 132.6 | 140.0 | 146.1 |
| 8 | 97.0 | 104.2 | 112.7 | 118.7 | 123.7 | 128.4 | 133.0 | 137.8 | 143.4 | 151.2 | 157.5 |
| 9 | 105.6 | 113.0 | 121.7 | 127.8 | 132.9 | 137.6 | 142.3 | 147.2 | 152.9 | 160.8 | 167.2 |
| 10 | 111.7 | 119.5 | 128.7 | 135.0 | 140.4 | 145.3 | 150.2 | 155.4 | 161.3 | 169.5 | 176.1 |
| 11 | 118.8 | 126.6 | 135.9 | 142.4 | 147.9 | 153.0 | 158.0 | 163.4 | 169.6 | 178.1 | 185.2 |
| 12 | 125.2 | 133.6 | 143.6 | 150.7 | 156.7 | 162.4 | 167.9 | 173.9 | 180.9 | 190.5 | 198.5 |
| 13 | 137.4 | 146.6 | 157.6 | 165.4 | 172.0 | 178.1 | 184.3 | 190.9 | 198.6 | 209.3 | 218.1 |
| 14 | 149.8 | 159.9 | 171.7 | 179.9 | 186.9 | 193.3 | 199.6 | 206.3 | 214.0 | 224.7 | 233.4 |
| 15 | 163.4 | 174.1 | 186.3 | 194.6 | 201.5 | 207.8 | 213.9 | 220.3 | 227.6 | 237.6 | 245.7 |
| 16 | 176.1 | 186.5 | 198.2 | 206.2 | 212.7 | 218.6 | 224.4 | 230.4 | 237.2 | 246.4 | 253.8 |
| 17 | 181.9 | 192.2 | 203.8 | 211.6 | 218.1 | 224.0 | 229.7 | 235.6 | 242.4 | 251.6 | 259.0 |
| 18 | 184.9 | 195.0 | 206.4 | 214.2 | 220.6 | 226.4 | 232.1 | 238.1 | 244.9 | 254.2 | 261.8 |
| Girls |  |  |  |  |  |  |  |  |  |  |  |
| 7 | 82.7 | 88.8 | 96.1 | 101.3 | 105.8 | 109.9 | 114.0 | 118.4 | 123.6 | 130.8 | 136.9 |
| 8 | 91.1 | 97.6 | 105.3 | 110.8 | 115.4 | 119.7 | 124.0 | 128.6 | 134.0 | 141.6 | 147.9 |
| 9 | 98.7 | 105.5 | 113.5 | 119.2 | 124.0 | 128.5 | 133.0 | 137.7 | 143.3 | 151.1 | 157.6 |
| 10 | 105.3 | 112.4 | 120.7 | 126.6 | 131.6 | 136.3 | 140.9 | 145.8 | 151.6 | 159.6 | 166.3 |
| 11 | 111.9 | 119.3 | 128.1 | 134.2 | 139.4 | 144.2 | 149.0 | 154.2 | 160.1 | 168.5 | 175.4 |
| 12 | 116.8 | 124.5 | 133.5 | 139.8 | 145.2 | 150.1 | 155.1 | 160.3 | 166.5 | 175.0 | 182.1 |
| 13 | 121.4 | 129.2 | 138.4 | 144.8 | 150.3 | 155.3 | 160.3 | 165.6 | 171.9 | 180.5 | 187.8 |
| 14 | 125.0 | 132.8 | 141.8 | 148.2 | 153.6 | 158.6 | 163.6 | 168.9 | 175.1 | 183.7 | 190.9 |
| 15 | 129.2 | 136.8 | 145.7 | 152.0 | 157.3 | 162.2 | 167.1 | 172.3 | 178.4 | 186.9 | 194.0 |
| 16 | 133.7 | 141.2 | 150.0 | 156.2 | 161.4 | 166.3 | 171.1 | 176.2 | 182.2 | 190.7 | 197.9 |
| 17 | 134.5 | 142.1 | 150.8 | 156.8 | 161.8 | 166.5 | 171.1 | 176.1 | 182.0 | 190.5 | 197.8 |
| 18 | 134.4 | 142.3 | 150.9 | 156.9 | 161.8 | 166.3 | 170.8 | 175.7 | 181.6 | 190.1 | 197.8 |

**Table S5** Standing long jump (cm) percentile values by age group and sex among Chinese children and adolescents aged 7–18 years based on normal weight population

| Age（years） | P_5_ | P_10_ | P_20_ | P_30_ | P_40_ | P_50_ | P_60_ | P_70_ | P_80_ | P_90_ | P_95_ |
| --- | --- | --- | --- | --- | --- | --- | --- | --- | --- | --- | --- |
| Boys |  |  |  |  |  |  |  |  |  |  |  |
| 7 | 89.7 | 96.7 | 104.9 | 110.6 | 115.3 | 119.6 | 123.9 | 128.4 | 133.6 | 140.7 | 146.5 |
| 8 | 100.2 | 107.4 | 115.7 | 121.6 | 126.4 | 130.9 | 135.3 | 140.0 | 145.4 | 152.9 | 159.0 |
| 9 | 109.7 | 117.1 | 125.6 | 131.5 | 136.5 | 141.0 | 145.5 | 150.2 | 155.7 | 163.2 | 169.3 |
| 10 | 117.0 | 124.7 | 133.6 | 139.7 | 144.9 | 149.6 | 154.2 | 159.0 | 164.6 | 172.3 | 178.5 |
| 11 | 123.6 | 131.4 | 140.5 | 146.9 | 152.2 | 157.1 | 161.9 | 166.9 | 172.8 | 180.9 | 187.6 |
| 12 | 130.1 | 138.5 | 148.4 | 155.3 | 161.1 | 166.5 | 171.9 | 177.5 | 184.1 | 193.3 | 200.8 |
| 13 | 141.7 | 150.9 | 161.7 | 169.3 | 175.7 | 181.7 | 187.6 | 194.0 | 201.3 | 211.6 | 220.1 |
| 14 | 154.9 | 164.7 | 176.1 | 184.1 | 190.8 | 196.9 | 203.0 | 209.4 | 216.8 | 227.1 | 235.6 |
| 15 | 168.0 | 178.4 | 190.2 | 198.2 | 204.9 | 210.9 | 216.8 | 222.9 | 230.0 | 239.6 | 247.4 |
| 16 | 180.6 | 190.6 | 201.9 | 209.5 | 215.7 | 221.4 | 226.9 | 232.6 | 239.2 | 248.1 | 255.3 |
| 17 | 186.4 | 196.3 | 207.4 | 214.9 | 221.2 | 226.8 | 232.3 | 238.0 | 244.5 | 253.4 | 260.6 |
| 18 | 189.1 | 198.8 | 209.8 | 217.3 | 223.5 | 229.1 | 234.6 | 240.4 | 247.0 | 256.0 | 263.4 |
| Girls |  |  |  |  |  |  |  |  |  |  |  |
| 7 | 83.3 | 89.5 | 96.8 | 102.0 | 106.4 | 110.5 | 114.6 | 119.0 | 124.2 | 131.3 | 137.4 |
| 8 | 92.2 | 98.7 | 106.4 | 111.9 | 116.5 | 120.8 | 125.1 | 129.7 | 135.0 | 142.5 | 148.7 |
| 9 | 100.1 | 106.9 | 114.9 | 120.6 | 125.4 | 129.8 | 134.2 | 139.0 | 144.5 | 152.2 | 158.6 |
| 10 | 107.1 | 114.2 | 122.5 | 128.3 | 133.3 | 137.9 | 142.4 | 147.3 | 153.0 | 160.9 | 167.5 |
| 11 | 113.7 | 121.1 | 129.8 | 135.9 | 141.0 | 145.8 | 150.5 | 155.6 | 161.5 | 169.6 | 176.5 |
| 12 | 118.4 | 126.1 | 135.1 | 141.4 | 146.7 | 151.7 | 156.6 | 161.8 | 167.8 | 176.3 | 183.3 |
| 13 | 122.7 | 130.5 | 139.6 | 146.1 | 151.5 | 156.5 | 161.5 | 166.8 | 172.9 | 181.5 | 188.7 |
| 14 | 126.1 | 133.8 | 142.9 | 149.3 | 154.7 | 159.6 | 164.6 | 169.8 | 176.0 | 184.5 | 191.6 |
| 15 | 130.1 | 137.7 | 146.6 | 152.9 | 158.2 | 163.1 | 167.9 | 173.1 | 179.1 | 187.6 | 194.6 |
| 16 | 134.5 | 142.1 | 150.9 | 157.0 | 162.2 | 167.0 | 171.8 | 176.9 | 182.9 | 191.3 | 198.4 |
| 17 | 135.3 | 142.8 | 151.5 | 157.5 | 162.5 | 167.1 | 171.7 | 176.7 | 182.5 | 190.9 | 198.2 |
| 18 | 135.1 | 142.9 | 151.6 | 157.4 | 162.3 | 166.8 | 171.3 | 176.1 | 181.9 | 190.5 | 198.1 |

**Table S6** 50-m dash (s) percentile values by age group and sex among Chinese children and adolescents aged 7–18 years based on total population

| Age（years） | P_5_ | P_10_ | P_20_ | P_30_ | P_40_ | P_50_ | P_60_ | P_70_ | P_80_ | P_90_ | P_95_ | |
| --- | --- | --- | --- | --- | --- | --- | --- | --- | --- | --- | --- | --- |
| Boys |  |  |  |  |  |  |  |  |  |  |  | |
| 7 | 9.64 | 9.98 | 10.41 | 10.73 | 11.00 | 11.27 | 11.55 | 11.86 | 12.25 | 12.85 | 13.42 |  |
| 8 | 9.23 | 9.52 | 9.88 | 10.15 | 10.39 | 10.62 | 10.87 | 11.15 | 11.51 | 12.07 | 12.61 |  |
| 9 | 8.84 | 9.10 | 9.43 | 9.68 | 9.91 | 10.12 | 10.36 | 10.62 | 10.95 | 11.47 | 11.97 |  |
| 10 | 8.55 | 8.80 | 9.11 | 9.35 | 9.57 | 9.78 | 10.00 | 10.26 | 10.58 | 11.09 | 11.58 |  |
| 11 | 8.25 | 8.50 | 8.81 | 9.05 | 9.26 | 9.47 | 9.69 | 9.94 | 10.25 | 10.74 | 11.20 |  |
| 12 | 7.90 | 8.15 | 8.46 | 8.71 | 8.92 | 9.14 | 9.36 | 9.62 | 9.95 | 10.45 | 10.93 |  |
| 13 | 7.42 | 7.67 | 7.99 | 8.23 | 8.45 | 8.66 | 8.89 | 9.14 | 9.46 | 9.95 | 10.41 |  |
| 14 | 7.09 | 7.30 | 7.58 | 7.79 | 7.99 | 8.18 | 8.39 | 8.64 | 8.95 | 9.46 | 9.97 |  |
| 15 | 6.87 | 7.05 | 7.29 | 7.48 | 7.65 | 7.82 | 8.01 | 8.23 | 8.51 | 8.97 | 9.42 |  |
| 16 | 6.75 | 6.93 | 7.15 | 7.33 | 7.49 | 7.65 | 7.82 | 8.01 | 8.27 | 8.67 | 9.08 |  |
| 17 | 6.66 | 6.83 | 7.05 | 7.22 | 7.37 | 7.53 | 7.69 | 7.89 | 8.14 | 8.56 | 8.98 |  |
| 18 | 6.63 | 6.80 | 7.02 | 7.20 | 7.35 | 7.51 | 7.67 | 7.87 | 8.13 | 8.55 | 8.99 |  |
| Girls |  |  |  |  |  |  |  |  |  |  |  |  |
| 7 | 10.19 | 10.51 | 10.91 | 11.21 | 11.48 | 11.74 | 12.01 | 12.32 | 12.72 | 13.34 | 13.94 |  |
| 8 | 9.69 | 9.98 | 10.34 | 10.61 | 10.85 | 11.08 | 11.33 | 11.61 | 11.96 | 12.50 | 13.02 |  |
| 9 | 9.28 | 9.55 | 9.89 | 10.14 | 10.36 | 10.58 | 10.81 | 11.07 | 11.39 | 11.90 | 12.37 |  |
| 10 | 8.94 | 9.21 | 9.54 | 9.78 | 10.00 | 10.21 | 10.43 | 10.68 | 11.00 | 11.49 | 11.95 |  |
| 11 | 8.63 | 8.90 | 9.22 | 9.46 | 9.67 | 9.88 | 10.10 | 10.35 | 10.66 | 11.15 | 11.61 |  |
| 12 | 8.41 | 8.67 | 8.99 | 9.23 | 9.45 | 9.66 | 9.87 | 10.12 | 10.44 | 10.93 | 11.40 |  |
| 13 | 8.29 | 8.55 | 8.87 | 9.12 | 9.33 | 9.54 | 9.76 | 10.01 | 10.33 | 10.83 | 11.31 |  |
| 14 | 8.21 | 8.48 | 8.80 | 9.04 | 9.26 | 9.47 | 9.70 | 9.95 | 10.28 | 10.79 | 11.30 |  |
| 15 | 8.17 | 8.44 | 8.76 | 9.01 | 9.23 | 9.45 | 9.67 | 9.94 | 10.27 | 10.80 | 11.33 |  |
| 16 | 8.18 | 8.44 | 8.78 | 9.03 | 9.25 | 9.47 | 9.71 | 9.97 | 10.32 | 10.87 | 11.42 |  |
| 17 | 8.17 | 8.45 | 8.78 | 9.04 | 9.27 | 9.49 | 9.73 | 10.01 | 10.37 | 10.95 | 11.52 |  |
| 18 | 8.21 | 8.48 | 8.83 | 9.09 | 9.32 | 9.55 | 9.80 | 10.08 | 10.45 | 11.05 | 11.65 |  |

**Table S7** 50-m dash (s) percentile values by age group and sex among Chinese children and adolescents aged 7–18 years based on normal weight population

| Age（years） | P_5_ | P_10_ | P_20_ | P_30_ | P_40_ | P_50_ | P_60_ | P_70_ | P_80_ | P_90_ | P_95_ | |
| --- | --- | --- | --- | --- | --- | --- | --- | --- | --- | --- | --- | --- |
| Boys |  |  |  |  |  |  |  |  |  |  |  | |
| 7 | 9.63 | 9.95 | 10.34 | 10.64 | 10.91 | 11.16 | 11.44 | 11.74 | 12.14 | 12.75 | 13.34 |  |
| 8 | 9.16 | 9.44 | 9.79 | 10.05 | 10.28 | 10.51 | 10.75 | 11.01 | 11.35 | 11.88 | 12.39 |  |
| 9 | 8.77 | 9.02 | 9.34 | 9.57 | 9.78 | 9.98 | 10.20 | 10.44 | 10.74 | 11.21 | 11.66 |  |
| 10 | 8.46 | 8.70 | 9.00 | 9.22 | 9.41 | 9.61 | 9.81 | 10.03 | 10.32 | 10.77 | 11.19 |  |
| 11 | 8.17 | 8.40 | 8.69 | 8.91 | 9.10 | 9.28 | 9.48 | 9.71 | 9.99 | 10.43 | 10.85 |  |
| 12 | 7.85 | 8.08 | 8.37 | 8.59 | 8.78 | 8.97 | 9.17 | 9.40 | 9.70 | 10.16 | 10.61 |  |
| 13 | 7.41 | 7.64 | 7.92 | 8.14 | 8.34 | 8.53 | 8.73 | 8.97 | 9.27 | 9.76 | 10.25 |  |
| 14 | 7.05 | 7.25 | 7.52 | 7.72 | 7.90 | 8.08 | 8.27 | 8.50 | 8.78 | 9.25 | 9.71 |  |
| 15 | 6.82 | 7.01 | 7.25 | 7.43 | 7.59 | 7.75 | 7.92 | 8.12 | 8.38 | 8.79 | 9.20 |  |
| 16 | 6.72 | 6.89 | 7.11 | 7.28 | 7.43 | 7.58 | 7.74 | 7.92 | 8.16 | 8.55 | 8.94 |  |
| 17 | 6.62 | 6.79 | 7.01 | 7.17 | 7.32 | 7.47 | 7.63 | 7.82 | 8.06 | 8.45 | 8.85 |  |
| 18 | 6.60 | 6.77 | 6.98 | 7.15 | 7.30 | 7.45 | 7.61 | 7.79 | 8.04 | 8.45 | 8.86 |  |
| Girls |  |  |  |  |  |  |  |  |  |  |  |  |
| 7 | 10.15 | 10.47 | 10.86 | 11.16 | 11.42 | 11.68 | 11.95 | 12.26 | 12.65 | 13.25 | 13.82 |  |
| 8 | 9.65 | 9.94 | 10.30 | 10.56 | 10.80 | 11.03 | 11.28 | 11.55 | 11.90 | 12.43 | 12.94 |  |
| 9 | 9.23 | 9.50 | 9.84 | 10.08 | 10.30 | 10.52 | 10.74 | 11.00 | 11.31 | 11.81 | 12.28 |  |
| 10 | 8.90 | 9.16 | 9.48 | 9.72 | 9.93 | 10.14 | 10.35 | 10.60 | 10.90 | 11.38 | 11.83 |  |
| 11 | 8.60 | 8.85 | 9.17 | 9.41 | 9.61 | 9.82 | 10.03 | 10.27 | 10.58 | 11.05 | 11.51 |  |
| 12 | 8.38 | 8.64 | 8.95 | 9.19 | 9.40 | 9.60 | 9.81 | 10.06 | 10.37 | 10.85 | 11.31 |  |
| 13 | 8.26 | 8.52 | 8.84 | 9.07 | 9.28 | 9.49 | 9.71 | 9.95 | 10.27 | 10.76 | 11.25 |  |
| 14 | 8.19 | 8.45 | 8.77 | 9.01 | 9.22 | 9.43 | 9.65 | 9.91 | 10.23 | 10.74 | 11.25 |  |
| 15 | 8.15 | 8.41 | 8.74 | 8.98 | 9.20 | 9.41 | 9.64 | 9.90 | 10.23 | 10.76 | 11.28 |  |
| 16 | 8.15 | 8.42 | 8.75 | 9.00 | 9.22 | 9.44 | 9.67 | 9.94 | 10.28 | 10.84 | 11.39 |  |
| 17 | 8.15 | 8.43 | 8.76 | 9.02 | 9.24 | 9.47 | 9.71 | 9.98 | 10.34 | 10.91 | 11.49 |  |
| 18 | 8.19 | 8.47 | 8.81 | 9.07 | 9.30 | 9.53 | 9.78 | 10.06 | 10.42 | 11.02 | 11.63 |  |

**Table S8** Sit-and-reach (cm) percentile values by age group and sex among Chinese children and adolescents aged 7–18 years based on total population

| Age（years） | P_5_ | P_10_ | P_20_ | P_30_ | P_40_ | P_50_ | P_60_ | P_70_ | P_80_ | P_90_ | P_95_ |
| --- | --- | --- | --- | --- | --- | --- | --- | --- | --- | --- | --- |
| Boys |  |  |  |  |  |  |  |  |  |  |  |
| 7 | -2.96 | -0.77 | 1.80 | 3.60 | 5.11 | 6.49 | 7.86 | 9.31 | 10.97 | 13.24 | 15.08 |
| 8 | -3.30 | -1.06 | 1.54 | 3.36 | 4.88 | 6.27 | 7.64 | 9.10 | 10.78 | 13.10 | 14.99 |
| 9 | -3.89 | -1.58 | 1.09 | 2.94 | 4.48 | 5.88 | 7.26 | 8.74 | 10.46 | 12.84 | 14.81 |
| 10 | -5.15 | -2.70 | 0.11 | 2.04 | 3.62 | 5.06 | 6.49 | 8.01 | 9.80 | 12.30 | 14.38 |
| 11 | -6.00 | -3.42 | -0.47 | 1.53 | 3.18 | 4.66 | 6.13 | 7.71 | 9.57 | 12.18 | 14.36 |
| 12 | -6.63 | -3.93 | -0.86 | 1.22 | 2.91 | 4.44 | 5.95 | 7.58 | 9.50 | 12.21 | 14.48 |
| 13 | -6.31 | -3.48 | -0.27 | 1.90 | 3.68 | 5.28 | 6.85 | 8.55 | 10.56 | 13.37 | 15.72 |
| 14 | -5.57 | -2.58 | 0.81 | 3.12 | 5.01 | 6.72 | 8.39 | 10.19 | 12.29 | 15.21 | 17.63 |
| 15 | -4.25 | -1.16 | 2.35 | 4.76 | 6.73 | 8.53 | 10.29 | 12.14 | 14.30 | 17.25 | 19.65 |
| 16 | -2.80 | 0.28 | 3.80 | 6.23 | 8.23 | 10.06 | 11.84 | 13.71 | 15.84 | 18.73 | 21.05 |
| 17 | -2.23 | 0.89 | 4.45 | 6.90 | 8.93 | 10.78 | 12.59 | 14.46 | 16.58 | 19.42 | 21.67 |
| 18 | -1.91 | 1.25 | 4.86 | 7.35 | 9.41 | 11.29 | 13.12 | 15.00 | 17.12 | 19.93 | 22.14 |
| Girls |  |  |  |  |  |  |  |  |  |  |  |
| 7 | 1.45 | 3.39 | 5.76 | 7.49 | 8.98 | 10.38 | 11.75 | 13.15 | 14.69 | 16.68 | 18.22 |
| 8 | 1.20 | 3.24 | 5.72 | 7.51 | 9.06 | 10.49 | 11.91 | 13.36 | 14.98 | 17.09 | 18.73 |
| 9 | 0.44 | 2.60 | 5.19 | 7.05 | 8.65 | 10.13 | 11.59 | 13.10 | 14.79 | 17.03 | 18.78 |
| 10 | -0.40 | 1.88 | 4.60 | 6.54 | 8.20 | 9.74 | 11.25 | 12.82 | 14.59 | 16.95 | 18.82 |
| 11 | -1.04 | 1.38 | 4.26 | 6.31 | 8.05 | 9.66 | 11.24 | 12.89 | 14.75 | 17.25 | 19.24 |
| 12 | -1.52 | 1.06 | 4.10 | 6.25 | 8.07 | 9.75 | 11.39 | 13.11 | 15.06 | 17.67 | 19.75 |
| 13 | -1.26 | 1.41 | 4.56 | 6.77 | 8.63 | 10.35 | 12.04 | 13.79 | 15.78 | 18.43 | 20.54 |
| 14 | -0.54 | 2.25 | 5.51 | 7.80 | 9.73 | 11.50 | 13.23 | 15.02 | 17.04 | 19.73 | 21.86 |
| 15 | 0.31 | 3.15 | 6.45 | 8.76 | 10.70 | 12.48 | 14.22 | 16.01 | 18.02 | 20.67 | 22.76 |
| 16 | 1.23 | 4.05 | 7.32 | 9.61 | 11.53 | 13.30 | 15.01 | 16.77 | 18.74 | 21.32 | 23.34 |
| 17 | 1.64 | 4.47 | 7.75 | 10.04 | 11.96 | 13.71 | 15.42 | 17.16 | 19.10 | 21.62 | 23.59 |
| 18 | 1.85 | 4.71 | 8.00 | 10.30 | 12.22 | 13.98 | 15.68 | 17.41 | 19.33 | 21.81 | 23.73 |

**Table S9** Sit-and-reach (cm) percentile values by age group and sex among Chinese children and adolescents aged 7–18 years based on normal weight population

| Age（years） | P_5_ | P_10_ | P_20_ | P_30_ | P_40_ | P_50_ | P_60_ | P_70_ | P_80_ | P_90_ | P_95_ |
| --- | --- | --- | --- | --- | --- | --- | --- | --- | --- | --- | --- |
| Boys |  |  |  |  |  |  |  |  |  |  |  |
| 7 | -2.77 | -0.58 | 1.94 | 3.68 | 5.11 | 6.41 | 7.69 | 9.08 | 10.72 | 13.02 | 14.95 |
| 8 | -3.06 | -0.83 | 1.75 | 3.52 | 4.98 | 6.30 | 7.61 | 9.02 | 10.69 | 13.04 | 14.99 |
| 9 | -3.69 | -1.39 | 1.26 | 3.08 | 4.58 | 5.95 | 7.29 | 8.74 | 10.45 | 12.85 | 14.84 |
| 10 | -4.70 | -2.30 | 0.45 | 2.35 | 3.91 | 5.33 | 6.73 | 8.23 | 10.00 | 12.46 | 14.51 |
| 11 | -5.51 | -3.01 | -0.13 | 1.84 | 3.47 | 4.95 | 6.41 | 7.97 | 9.80 | 12.35 | 14.46 |
| 12 | -6.18 | -3.57 | -0.58 | 1.47 | 3.16 | 4.70 | 6.21 | 7.82 | 9.70 | 12.32 | 14.47 |
| 13 | -6.08 | -3.33 | -0.18 | 1.98 | 3.75 | 5.37 | 6.95 | 8.63 | 10.60 | 13.30 | 15.53 |
| 14 | -5.26 | -2.32 | 1.03 | 3.32 | 5.20 | 6.91 | 8.59 | 10.36 | 12.42 | 15.25 | 17.56 |
| 15 | -3.98 | -0.90 | 2.60 | 4.99 | 6.96 | 8.74 | 10.49 | 12.33 | 14.45 | 17.35 | 19.70 |
| 16 | -2.62 | 0.51 | 4.06 | 6.49 | 8.48 | 10.29 | 12.05 | 13.90 | 16.03 | 18.92 | 21.25 |
| 17 | -1.89 | 1.26 | 4.83 | 7.26 | 9.25 | 11.06 | 12.82 | 14.67 | 16.77 | 19.62 | 21.90 |
| 18 | -1.33 | 1.85 | 5.43 | 7.87 | 9.87 | 11.69 | 13.45 | 15.28 | 17.37 | 20.17 | 22.41 |
| Girls |  |  |  |  |  |  |  |  |  |  |  |
| 7 | 1.39 | 3.38 | 5.80 | 7.56 | 9.08 | 10.50 | 11.88 | 13.27 | 14.78 | 16.68 | 18.13 |
| 8 | 1.17 | 3.24 | 5.73 | 7.53 | 9.09 | 10.54 | 11.95 | 13.40 | 15.00 | 17.08 | 18.68 |
| 9 | 0.46 | 2.61 | 5.20 | 7.07 | 8.66 | 10.15 | 11.61 | 13.12 | 14.82 | 17.06 | 18.82 |
| 10 | -0.21 | 2.01 | 4.69 | 6.60 | 8.24 | 9.76 | 11.26 | 12.83 | 14.62 | 17.02 | 18.95 |
| 11 | -0.83 | 1.52 | 4.33 | 6.34 | 8.03 | 9.61 | 11.17 | 12.82 | 14.71 | 17.29 | 19.38 |
| 12 | -1.32 | 1.16 | 4.11 | 6.21 | 7.98 | 9.63 | 11.25 | 12.97 | 14.95 | 17.66 | 19.86 |
| 13 | -1.05 | 1.53 | 4.60 | 6.77 | 8.61 | 10.31 | 11.98 | 13.74 | 15.76 | 18.48 | 20.68 |
| 14 | -0.42 | 2.36 | 5.61 | 7.90 | 9.83 | 11.60 | 13.34 | 15.13 | 17.16 | 19.86 | 21.99 |
| 15 | 0.29 | 3.20 | 6.56 | 8.91 | 10.87 | 12.67 | 14.41 | 16.19 | 18.17 | 20.76 | 22.77 |
| 16 | 1.16 | 4.07 | 7.42 | 9.75 | 11.70 | 13.49 | 15.21 | 16.96 | 18.89 | 21.40 | 23.33 |
| 17 | 1.60 | 4.49 | 7.83 | 10.15 | 12.09 | 13.86 | 15.58 | 17.32 | 19.25 | 21.73 | 23.66 |
| 18 | 1.90 | 4.76 | 8.06 | 10.36 | 12.29 | 14.06 | 15.77 | 17.51 | 19.43 | 21.93 | 23.86 |

**Table S10** Grip strength (kg) percentile values by age group and sex among Chinese children and adolescents aged 7–18 years based on total population

| Age（years） | P_5_ | P_10_ | P_20_ | P_30_ | P_40_ | P_50_ | P_60_ | P_70_ | P_80_ | P_90_ | P_95_ |
| --- | --- | --- | --- | --- | --- | --- | --- | --- | --- | --- | --- |
| Boys |  |  |  |  |  |  |  |  |  |  |  |
| 7 | 5.56 | 6.51 | 7.61 | 8.39 | 9.05 | 9.67 | 10.30 | 10.99 | 11.81 | 13.03 | 14.12 |
| 8 | 7.01 | 8.00 | 9.17 | 10.00 | 10.70 | 11.37 | 12.04 | 12.78 | 13.68 | 15.00 | 16.18 |
| 9 | 8.44 | 9.52 | 10.80 | 11.71 | 12.49 | 13.23 | 13.98 | 14.81 | 15.81 | 17.29 | 18.62 |
| 10 | 9.86 | 11.01 | 12.38 | 13.37 | 14.22 | 15.03 | 15.85 | 16.76 | 17.88 | 19.53 | 21.03 |
| 11 | 11.40 | 12.64 | 14.14 | 15.24 | 16.20 | 17.12 | 18.07 | 19.13 | 20.44 | 22.42 | 24.24 |
| 12 | 13.16 | 14.62 | 16.45 | 17.83 | 19.05 | 20.23 | 21.47 | 22.87 | 24.63 | 27.31 | 29.81 |
| 13 | 16.04 | 17.91 | 20.29 | 22.09 | 23.70 | 25.27 | 26.89 | 28.72 | 30.97 | 34.35 | 37.40 |
| 14 | 19.83 | 22.18 | 25.08 | 27.23 | 29.11 | 30.90 | 32.73 | 34.74 | 37.18 | 40.71 | 43.79 |
| 15 | 24.12 | 26.65 | 29.69 | 31.88 | 33.77 | 35.55 | 37.35 | 39.32 | 41.69 | 45.11 | 48.10 |
| 16 | 27.21 | 29.84 | 32.90 | 35.05 | 36.87 | 38.59 | 40.32 | 42.22 | 44.53 | 47.95 | 51.04 |
| 17 | 29.28 | 31.97 | 35.05 | 37.20 | 39.02 | 40.72 | 42.45 | 44.35 | 46.67 | 50.16 | 53.38 |
| 18 | 30.62 | 33.34 | 36.45 | 38.62 | 40.45 | 42.18 | 43.92 | 45.85 | 48.21 | 51.77 | 55.07 |
| Girls |  |  |  |  |  |  |  |  |  |  |  |
| 7 | 4.82 | 5.62 | 6.57 | 7.25 | 7.84 | 8.39 | 8.95 | 9.55 | 10.29 | 11.35 | 12.28 |
| 8 | 5.93 | 6.83 | 7.90 | 8.65 | 9.30 | 9.90 | 10.52 | 11.19 | 11.99 | 13.18 | 14.23 |
| 9 | 7.25 | 8.25 | 9.42 | 10.25 | 10.96 | 11.63 | 12.31 | 13.06 | 13.98 | 15.33 | 16.56 |
| 10 | 8.63 | 9.70 | 10.98 | 11.91 | 12.70 | 13.46 | 14.23 | 15.08 | 16.13 | 17.70 | 19.12 |
| 11 | 10.43 | 11.64 | 13.12 | 14.20 | 15.14 | 16.04 | 16.97 | 17.99 | 19.24 | 21.11 | 22.79 |
| 12 | 12.24 | 13.63 | 15.33 | 16.59 | 17.67 | 18.71 | 19.78 | 20.96 | 22.39 | 24.49 | 26.36 |
| 13 | 14.14 | 15.66 | 17.50 | 18.85 | 20.01 | 21.11 | 22.23 | 23.47 | 24.95 | 27.12 | 29.02 |
| 14 | 15.65 | 17.24 | 19.15 | 20.52 | 21.70 | 22.82 | 23.94 | 25.17 | 26.65 | 28.80 | 30.67 |
| 15 | 16.56 | 18.21 | 20.16 | 21.56 | 22.75 | 23.87 | 25.01 | 26.25 | 27.74 | 29.91 | 31.83 |
| 16 | 17.26 | 18.95 | 20.94 | 22.35 | 23.56 | 24.70 | 25.85 | 27.12 | 28.65 | 30.90 | 32.93 |
| 17 | 17.62 | 19.37 | 21.40 | 22.84 | 24.06 | 25.21 | 26.38 | 27.66 | 29.21 | 31.52 | 33.61 |
| 18 | 17.81 | 19.63 | 21.73 | 23.21 | 24.46 | 25.64 | 26.83 | 28.13 | 29.70 | 32.03 | 34.12 |

**Table S11** Grip strength (kg) percentile values by age group and sex among Chinese children and adolescents aged 7–18 years based on normal weight population

| Age（years） | P_5_ | P_10_ | P_20_ | P_30_ | P_40_ | P_50_ | P_60_ | P_70_ | P_80_ | P_90_ | P_95_ |
| --- | --- | --- | --- | --- | --- | --- | --- | --- | --- | --- | --- |
| Boys |  |  |  |  |  |  |  |  |  |  |  |
| 7 | 5.48 | 6.40 | 7.46 | 8.20 | 8.82 | 9.41 | 9.99 | 10.62 | 11.38 | 12.48 | 13.45 |
| 8 | 6.89 | 7.85 | 8.97 | 9.75 | 10.41 | 11.03 | 11.66 | 12.33 | 13.15 | 14.35 | 15.42 |
| 9 | 8.35 | 9.38 | 10.59 | 11.43 | 12.15 | 12.83 | 13.51 | 14.26 | 15.17 | 16.51 | 17.72 |
| 10 | 9.76 | 10.83 | 12.09 | 12.99 | 13.77 | 14.50 | 15.24 | 16.06 | 17.06 | 18.54 | 19.88 |
| 11 | 11.22 | 12.37 | 13.76 | 14.77 | 15.64 | 16.48 | 17.34 | 18.29 | 19.46 | 21.22 | 22.83 |
| 12 | 12.98 | 14.35 | 16.07 | 17.34 | 18.46 | 19.55 | 20.68 | 21.94 | 23.51 | 25.88 | 28.06 |
| 13 | 15.88 | 17.67 | 19.92 | 21.62 | 23.11 | 24.56 | 26.06 | 27.73 | 29.78 | 32.82 | 35.54 |
| 14 | 19.79 | 22.03 | 24.79 | 26.82 | 28.59 | 30.27 | 31.99 | 33.86 | 36.12 | 39.39 | 42.23 |
| 15 | 24.31 | 26.72 | 29.60 | 31.69 | 33.48 | 35.17 | 36.88 | 38.75 | 40.99 | 44.23 | 47.05 |
| 16 | 27.47 | 30.00 | 32.91 | 34.95 | 36.68 | 38.31 | 39.95 | 41.75 | 43.94 | 47.19 | 50.14 |
| 17 | 29.69 | 32.32 | 35.30 | 37.35 | 39.09 | 40.71 | 42.35 | 44.14 | 46.34 | 49.65 | 52.72 |
| 18 | 30.86 | 33.57 | 36.63 | 38.74 | 40.51 | 42.17 | 43.85 | 45.69 | 47.96 | 51.38 | 54.57 |
| Girls |  |  |  |  |  |  |  |  |  |  |  |
| 7 | 4.79 | 5.59 | 6.53 | 7.20 | 7.76 | 8.29 | 8.83 | 9.41 | 10.10 | 11.11 | 11.99 |
| 8 | 5.91 | 6.81 | 7.85 | 8.58 | 9.20 | 9.78 | 10.37 | 11.01 | 11.79 | 12.93 | 13.95 |
| 9 | 7.16 | 8.14 | 9.28 | 10.08 | 10.76 | 11.39 | 12.04 | 12.75 | 13.61 | 14.88 | 16.04 |
| 10 | 8.52 | 9.58 | 10.82 | 11.70 | 12.46 | 13.17 | 13.89 | 14.68 | 15.65 | 17.07 | 18.36 |
| 11 | 10.31 | 11.51 | 12.95 | 13.99 | 14.88 | 15.73 | 16.59 | 17.53 | 18.67 | 20.35 | 21.85 |
| 12 | 12.24 | 13.61 | 15.27 | 16.47 | 17.50 | 18.48 | 19.48 | 20.58 | 21.90 | 23.84 | 25.54 |
| 13 | 14.18 | 15.65 | 17.43 | 18.71 | 19.81 | 20.86 | 21.92 | 23.08 | 24.48 | 26.51 | 28.30 |
| 14 | 15.73 | 17.27 | 19.11 | 20.42 | 21.55 | 22.62 | 23.70 | 24.88 | 26.29 | 28.36 | 30.18 |
| 15 | 16.61 | 18.22 | 20.11 | 21.47 | 22.62 | 23.70 | 24.80 | 26.00 | 27.45 | 29.57 | 31.45 |
| 16 | 17.27 | 18.96 | 20.94 | 22.33 | 23.52 | 24.63 | 25.76 | 26.99 | 28.48 | 30.68 | 32.65 |
| 17 | 17.58 | 19.33 | 21.36 | 22.78 | 23.98 | 25.11 | 26.25 | 27.50 | 29.01 | 31.25 | 33.27 |
| 18 | 17.81 | 19.61 | 21.69 | 23.15 | 24.39 | 25.55 | 26.72 | 28.00 | 29.54 | 31.82 | 33.85 |

**Table S12** Body muscle strength percentile values by age group and sex among Chinese boys aged 7–18 years based on total population

| Age（years） | P_5_ | P_10_ | P_20_ | P_30_ | P_40_ | P_50_ | P_60_ | P_70_ | P_80_ | P_90_ | P_95_ |
| --- | --- | --- | --- | --- | --- | --- | --- | --- | --- | --- | --- |
| Boys (oblique body pull-ups) | | | | | | | | | | | |
| 7 | 4.1 | 6.1 | 9.2 | 11.9 | 14.6 | 17.5 | 20.7 | 24.7 | 29.9 | 38.3 | 46.6 |
| 8 | 4.5 | 6.6 | 9.8 | 12.7 | 15.5 | 18.6 | 22.0 | 26.1 | 31.6 | 40.5 | 49.3 |
| 9 | 4.9 | 7.1 | 10.5 | 13.4 | 16.4 | 19.5 | 23.1 | 27.4 | 33.1 | 42.5 | 51.8 |
| 10 | 5.1 | 7.4 | 10.9 | 14.0 | 17.1 | 20.3 | 24.0 | 28.3 | 34.2 | 43.9 | 53.5 |
| 11 | 5.1 | 7.6 | 11.3 | 14.6 | 17.8 | 21.1 | 24.8 | 29.3 | 35.3 | 45.0 | 54.6 |
| 12 | 4.9 | 7.7 | 12.0 | 15.5 | 19.0 | 22.5 | 26.4 | 31.1 | 37.2 | 47.1 | 57.0 |
| Boys (pull-ups) | | | | | | | | | | | |
| 13 | 0.0 | 0.0 | 0.0 | 0.0 | 0.0 | 1.0 | 1.0 | 2.0 | 3.0 | 5.0 | 7.0 |
| 14 | 0.0 | 0.0 | 0.0 | 1.0 | 1.0 | 2.0 | 2.0 | 3.0 | 5.0 | 7.0 | 9.0 |
| 15 | 0.0 | 0.0 | 0.0 | 1.0 | 2.0 | 2.0 | 3.0 | 4.0 | 6.0 | 8.0 | 10.0 |
| 16 | 0.0 | 0.0 | 1.0 | 1.0 | 2.0 | 3.0 | 4.0 | 5.0 | 6.0 | 9.0 | 10.0 |
| 17 | 0.0 | 0.0 | 1.0 | 2.0 | 3.0 | 3.0 | 4.0 | 5.0 | 7.0 | 10.0 | 11.0 |
| 18 | 0.0 | 0.0 | 1.0 | 2.0 | 3.0 | 4.0 | 5.0 | 6.0 | 7.0 | 10.0 | 11.0 |
| Girls (1-min sit-ups) | | | | | | | | | | | |
| 7 | 2.5 | 5.1 | 9.0 | 12.2 | 15.1 | 17.9 | 20.8 | 23.8 | 27.5 | 32.5 | 36.6 |
| 8 | 4.5 | 7.8 | 12.1 | 15.4 | 18.1 | 20.6 | 23.2 | 26.0 | 29.4 | 34.2 | 38.4 |
| 9 | 6.6 | 10.3 | 15.1 | 18.5 | 21.2 | 23.6 | 26.0 | 28.8 | 32.1 | 37.1 | 41.3 |
| 10 | 8.3 | 12.4 | 17.3 | 20.7 | 23.4 | 25.7 | 28.1 | 30.8 | 34.1 | 39.1 | 43.3 |
| 11 | 10.3 | 14.5 | 19.3 | 22.5 | 25.1 | 27.4 | 29.7 | 32.3 | 35.6 | 40.4 | 44.7 |
| 12 | 11.7 | 15.7 | 20.4 | 23.4 | 25.9 | 28.1 | 30.2 | 32.7 | 35.8 | 40.4 | 44.6 |
| 13 | 12.1 | 16.1 | 20.6 | 23.7 | 26.1 | 28.3 | 30.4 | 32.8 | 35.9 | 40.4 | 44.5 |
| 14 | 12.7 | 16.7 | 21.4 | 24.6 | 27.1 | 29.5 | 31.8 | 34.3 | 37.5 | 42.1 | 46.1 |
| 15 | 13.5 | 17.5 | 22.3 | 25.6 | 28.3 | 30.8 | 33.3 | 36.0 | 39.3 | 43.9 | 47.9 |
| 16 | 14.5 | 18.4 | 23.0 | 26.3 | 29.0 | 31.6 | 34.1 | 36.8 | 40.1 | 44.6 | 48.4 |
| 17 | 15.0 | 18.8 | 23.3 | 26.5 | 29.2 | 31.7 | 34.2 | 36.9 | 40.1 | 44.5 | 48.2 |
| 18 | 15.0 | 18.8 | 23.2 | 26.3 | 28.9 | 31.2 | 33.6 | 36.2 | 39.2 | 43.6 | 47.2 |

**Table S13** Body muscle strength percentile values by age group and sex among Chinese boys aged 7–18 years based on normal weight population

| Age（years） | P_5_ | P_10_ | P_20_ | P_30_ | P_40_ | P_50_ | P_60_ | P_70_ | P_80_ | P_90_ | P_95_ |
| --- | --- | --- | --- | --- | --- | --- | --- | --- | --- | --- | --- |
| Boys (oblique body pull-ups) | | | | | | | | | | | |
| 7 | 4.6 | 6.6 | 9.8 | 12.7 | 15.5 | 18.4 | 21.8 | 25.8 | 31.1 | 39.7 | 48.0 |
| 8 | 5.3 | 7.5 | 10.9 | 13.8 | 16.7 | 19.8 | 23.3 | 27.5 | 33.1 | 42.1 | 50.8 |
| 9 | 6.0 | 8.3 | 11.8 | 14.9 | 17.9 | 21.1 | 24.7 | 29.1 | 34.9 | 44.4 | 53.6 |
| 10 | 6.5 | 9.0 | 12.7 | 15.9 | 19.1 | 22.4 | 26.1 | 30.6 | 36.6 | 46.4 | 56.0 |
| 11 | 6.7 | 9.4 | 13.4 | 16.8 | 20.1 | 23.6 | 27.4 | 32.0 | 38.1 | 48.1 | 57.9 |
| 12 | 6.5 | 9.6 | 14.0 | 17.7 | 21.2 | 24.8 | 28.7 | 33.4 | 39.6 | 49.8 | 59.9 |
| Boys (pull-ups) | | | | | | | | | | | |
| 13 | 0.0 | 0.0 | 0.0 | 0.0 | 1.0 | 1.0 | 2.0 | 3.0 | 4.0 | 6.0 | 8.0 |
| 14 | 0.0 | 0.0 | 0.0 | 1.0 | 1.0 | 2.0 | 3.0 | 4.0 | 5.0 | 7.0 | 10.0 |
| 15 | 0.0 | 0.0 | 1.0 | 1.0 | 2.0 | 3.0 | 4.0 | 5.0 | 6.0 | 9.0 | 11.0 |
| 16 | 0.0 | 0.0 | 1.0 | 2.0 | 3.0 | 3.0 | 4.0 | 5.0 | 7.0 | 9.0 | 11.0 |
| 17 | 0.0 | 0.0 | 1.0 | 2.0 | 3.0 | 4.0 | 5.0 | 6.0 | 8.0 | 10.0 | 12.0 |
| 18 | 0.0 | 0.0 | 1.0 | 2.0 | 3.0 | 4.0 | 5.0 | 6.0 | 8.0 | 10.0 | 12.0 |
| Girls (1-min sit-ups) | | | | | | | | | | | |
| 7 | 3.1 | 5.8 | 9.8 | 13.0 | 15.7 | 18.3 | 21.0 | 23.9 | 27.5 | 32.6 | 37.0 |
| 8 | 4.7 | 8.0 | 12.4 | 15.7 | 18.4 | 20.9 | 23.4 | 26.2 | 29.6 | 34.6 | 38.8 |
| 9 | 6.9 | 10.7 | 15.5 | 18.9 | 21.6 | 24.0 | 26.5 | 29.3 | 32.6 | 37.6 | 41.9 |
| 10 | 8.9 | 13.0 | 17.9 | 21.2 | 23.9 | 26.3 | 28.7 | 31.4 | 34.7 | 39.6 | 43.9 |
| 11 | 10.9 | 15.0 | 19.9 | 23.1 | 25.7 | 28.1 | 30.4 | 33.0 | 36.2 | 41.0 | 45.3 |
| 12 | 12.2 | 16.2 | 20.8 | 23.8 | 26.3 | 28.5 | 30.7 | 33.2 | 36.3 | 40.8 | 44.8 |
| 13 | 12.6 | 16.5 | 21.0 | 24.1 | 26.5 | 28.7 | 30.9 | 33.4 | 36.4 | 40.9 | 44.8 |
| 14 | 13.1 | 17.1 | 21.7 | 24.9 | 27.5 | 29.8 | 32.1 | 34.7 | 37.8 | 42.4 | 46.4 |
| 15 | 13.8 | 17.9 | 22.6 | 25.9 | 28.6 | 31.0 | 33.5 | 36.2 | 39.4 | 44.2 | 48.2 |
| 16 | 14.6 | 18.6 | 23.2 | 26.5 | 29.2 | 31.7 | 34.1 | 36.8 | 40.1 | 44.7 | 48.7 |
| 17 | 15.1 | 19.0 | 23.5 | 26.7 | 29.4 | 31.8 | 34.3 | 36.9 | 40.1 | 44.6 | 48.4 |
| 18 | 15.2 | 18.9 | 23.3 | 26.4 | 29.0 | 31.3 | 33.7 | 36.3 | 39.4 | 43.7 | 47.4 |

**Table S14** Endurance running (s) percentile values by age group and sex among Chinese children and adolescents aged 7–18 years based on total population

| Age（years） | P_5_ | P_10_ | P_20_ | P_30_ | P_40_ | P_50_ | P_60_ | P_70_ | P_80_ | P_90_ | P_95_ |
| --- | --- | --- | --- | --- | --- | --- | --- | --- | --- | --- | --- |
| Boys (eight 50-m shuttle runs) | | | | | | | | | | | |
| 7 | 115.39 | 119.58 | 124.80 | 128.71 | 132.21 | 135.65 | 139.29 | 143.47 | 148.82 | 157.36 | 165.78 |
| 8 | 111.00 | 115.23 | 120.48 | 124.43 | 127.95 | 131.41 | 135.07 | 139.27 | 144.65 | 153.23 | 161.69 |
| 9 | 106.56 | 110.80 | 116.08 | 120.03 | 123.57 | 127.04 | 130.71 | 134.92 | 140.32 | 148.92 | 157.39 |
| 10 | 102.27 | 106.46 | 111.70 | 115.64 | 119.17 | 122.64 | 126.31 | 130.52 | 135.91 | 144.49 | 152.93 |
| 11 | 97.91 | 102.03 | 107.21 | 111.14 | 114.67 | 118.15 | 121.84 | 126.09 | 131.54 | 140.24 | 148.80 |
| 12 | 93.17 | 97.24 | 102.40 | 106.34 | 109.91 | 113.44 | 117.23 | 121.61 | 127.28 | 136.44 | 145.60 |
| Boys (1000-m endurance running) | | | | | | | | | | | |
| 13 | 240.00 | 252.88 | 269.51 | 282.24 | 293.63 | 304.73 | 316.41 | 329.71 | 346.45 | 372.01 | 395.39 |
| 14 | 229.81 | 239.88 | 253.55 | 264.62 | 275.03 | 285.60 | 296.91 | 309.70 | 325.55 | 349.28 | 370.66 |
| 15 | 219.35 | 228.20 | 240.21 | 249.92 | 259.05 | 268.33 | 278.33 | 289.76 | 304.16 | 326.23 | 346.63 |
| 16 | 217.53 | 225.99 | 237.11 | 245.80 | 253.71 | 261.56 | 269.95 | 279.63 | 292.03 | 311.47 | 329.89 |
| 17 | 216.53 | 224.82 | 235.79 | 244.41 | 252.31 | 260.18 | 268.57 | 278.21 | 290.45 | 309.42 | 327.17 |
| 18 | 214.45 | 222.83 | 233.94 | 242.69 | 250.72 | 258.74 | 267.29 | 277.06 | 289.41 | 308.40 | 326.02 |
| Girls (eight 50-m shuttle runs) | | | | | | | | | | | |
| 7 | 121.19 | 125.23 | 130.13 | 133.73 | 136.90 | 139.97 | 143.18 | 146.82 | 151.43 | 158.67 | 165.70 |
| 8 | 115.51 | 119.82 | 125.04 | 128.89 | 132.28 | 135.57 | 139.02 | 142.95 | 147.93 | 155.83 | 163.55 |
| 9 | 111.86 | 116.06 | 121.13 | 124.84 | 128.09 | 131.24 | 134.54 | 138.27 | 143.01 | 150.49 | 157.78 |
| 10 | 107.66 | 111.81 | 116.79 | 120.43 | 123.61 | 126.70 | 129.92 | 133.58 | 138.21 | 145.54 | 152.71 |
| 11 | 103.21 | 107.23 | 112.04 | 115.54 | 118.61 | 121.57 | 124.67 | 128.19 | 132.68 | 139.83 | 146.91 |
| 12 | 99.94 | 104.09 | 109.02 | 112.61 | 115.75 | 118.81 | 122.02 | 125.71 | 130.48 | 138.27 | 146.25 |
| Girls (800-m endurance running) | | | | | | | | | | | |
| 13 | 214.59 | 225.14 | 238.68 | 249.02 | 258.26 | 267.26 | 276.62 | 287.06 | 299.89 | 318.85 | 335.61 |
| 14 | 211.01 | 219.98 | 231.78 | 241.01 | 249.44 | 257.83 | 266.76 | 276.98 | 289.94 | 309.96 | 328.64 |
| 15 | 209.60 | 217.69 | 228.38 | 236.80 | 244.55 | 252.32 | 260.67 | 270.33 | 282.76 | 302.42 | 321.34 |
| 16 | 213.19 | 221.18 | 231.58 | 239.66 | 247.02 | 254.33 | 262.10 | 271.02 | 282.36 | 300.04 | 316.77 |
| 17 | 215.62 | 224.04 | 234.78 | 242.98 | 250.36 | 257.61 | 265.25 | 273.93 | 284.87 | 301.74 | 317.54 |
| 18 | 214.69 | 223.25 | 234.00 | 242.12 | 249.40 | 256.53 | 264.07 | 272.67 | 283.60 | 300.75 | 317.23 |

**Table S15** Endurance running (s) percentile values by age group and sex among Chinese children and adolescents aged 7–18 years based on normal weight population

| Age（years） | P_5_ | P_10_ | P_20_ | P_30_ | P_40_ | P_50_ | P_60_ | P_70_ | P_80_ | P_90_ | P_95_ |
| --- | --- | --- | --- | --- | --- | --- | --- | --- | --- | --- | --- |
| Boys (eight 50-m shuttle runs) | | | | | | | | | | | |
| 7 | 114.89 | 118.94 | 123.87 | 127.52 | 130.75 | 133.91 | 137.26 | 141.12 | 146.10 | 154.21 | 162.45 |
| 8 | 109.96 | 113.96 | 118.81 | 122.40 | 125.57 | 128.67 | 131.94 | 135.69 | 140.52 | 148.32 | 156.16 |
| 9 | 105.27 | 109.19 | 113.96 | 117.48 | 120.59 | 123.62 | 126.81 | 130.46 | 135.14 | 142.64 | 150.12 |
| 10 | 100.89 | 104.74 | 109.41 | 112.86 | 115.91 | 118.88 | 122.01 | 125.58 | 130.15 | 137.44 | 144.66 |
| 11 | 96.60 | 100.38 | 105.00 | 108.42 | 111.45 | 114.42 | 117.54 | 121.12 | 125.71 | 133.09 | 140.45 |
| 12 | 92.21 | 95.97 | 100.58 | 104.01 | 107.07 | 110.08 | 113.28 | 116.97 | 121.78 | 129.67 | 137.77 |
| Boys (1000-m endurance running) | | | | | | | | | | | |
| 13 | 236.39 | 248.56 | 264.23 | 276.18 | 286.85 | 297.22 | 308.10 | 320.44 | 335.92 | 359.41 | 380.78 |
| 14 | 227.78 | 237.30 | 250.13 | 260.44 | 270.07 | 279.81 | 290.19 | 301.93 | 316.49 | 338.32 | 358.01 |
| 15 | 217.13 | 225.70 | 237.14 | 246.24 | 254.66 | 263.12 | 272.20 | 282.62 | 295.85 | 316.36 | 335.57 |
| 16 | 216.31 | 224.37 | 234.85 | 242.92 | 250.20 | 257.35 | 264.97 | 273.81 | 285.24 | 303.35 | 320.71 |
| 17 | 214.96 | 222.80 | 233.16 | 241.28 | 248.72 | 256.12 | 264.03 | 273.11 | 284.66 | 302.62 | 319.46 |
| 18 | 212.86 | 220.93 | 231.58 | 239.93 | 247.57 | 255.18 | 263.26 | 272.50 | 284.15 | 302.05 | 318.62 |
| Girls (eight 50-m shuttle runs) | | | | | | | | | | | |
| 7 | 120.67 | 124.62 | 129.40 | 132.90 | 135.98 | 138.97 | 142.09 | 145.64 | 150.15 | 157.27 | 164.24 |
| 8 | 114.72 | 118.93 | 124.02 | 127.76 | 131.04 | 134.24 | 137.59 | 141.40 | 146.26 | 154.00 | 161.64 |
| 9 | 111.16 | 115.23 | 120.11 | 123.67 | 126.78 | 129.80 | 132.95 | 136.52 | 141.06 | 148.25 | 155.31 |
| 10 | 107.00 | 110.98 | 115.72 | 119.16 | 122.16 | 125.06 | 128.08 | 131.51 | 135.86 | 142.75 | 149.52 |
| 11 | 102.50 | 106.39 | 111.01 | 114.34 | 117.24 | 120.05 | 122.98 | 126.31 | 130.56 | 137.35 | 144.13 |
| 12 | 99.35 | 103.37 | 108.11 | 111.52 | 114.51 | 117.40 | 120.45 | 123.95 | 128.48 | 135.94 | 143.69 |
| Girls (800-m endurance running) | | | | | | | | | | | |
| 13 | 213.08 | 223.56 | 236.95 | 247.12 | 256.17 | 264.96 | 274.07 | 284.21 | 296.61 | 314.87 | 330.94 |
| 14 | 210.02 | 218.71 | 230.13 | 239.04 | 247.19 | 255.28 | 263.91 | 273.79 | 286.32 | 305.75 | 323.96 |
| 15 | 208.37 | 216.23 | 226.57 | 234.69 | 242.14 | 249.60 | 257.60 | 266.85 | 278.75 | 297.58 | 315.74 |
| 16 | 212.21 | 220.08 | 230.27 | 238.16 | 245.32 | 252.42 | 259.97 | 268.63 | 279.64 | 296.85 | 313.20 |
| 17 | 215.00 | 223.28 | 233.80 | 241.81 | 249.00 | 256.07 | 263.53 | 272.01 | 282.74 | 299.37 | 315.07 |
| 18 | 214.18 | 222.67 | 233.28 | 241.29 | 248.44 | 255.45 | 262.85 | 271.29 | 282.01 | 298.85 | 315.08 |
